# Selective Effects of Manual Massage and Foam Rolling on Perceived Recovery and Performance: Current Knowledge and Future Directions Toward Robotic Massages

**DOI:** 10.3389/fphys.2020.598898

**Published:** 2020-12-21

**Authors:** Yann Kerautret, Franck Di Rienzo, Carole Eyssautier, Aymeric Guillot

**Affiliations:** ^1^Univ Lyon, Université Claude Bernard Lyon 1, Laboratoire Interuniversitaire de Biologie de la Motricité EA 7424, Villeurbanne Cedex, France; ^2^Capsix Robotics, Lyon, France

**Keywords:** physiotherapy, manual massage, self-myofascial release, cobots, foam rolling, robotic, motor control

## Abstract

Manual massage and foam rolling are commonly used by athletes for warm-up and recovery, as well as by healthy individuals for well-being. Manual massage is an ancient practice requiring the intervention of an experienced physiotherapist, while foam rolling is a more recent self-administered technique. These two topics have been largely studied in isolation from each other. In the present review, we first provide a deep quantitative literature analysis to gather the beneficial effects of each technique through an integrative account, as well as their psychometric and neurophysiological evaluations. We then conceptually consider the motor control strategies induced by each type of massage. During manual massage, the person remains passive, lying on the massage table, and receives unanticipated manual pressure by the physiotherapist, hence resulting in a retroactive mode of action control with an ongoing central integration of proprioceptive feedback. In contrast, while performing foam rolling, the person directly exerts pressures through voluntary actions to manipulate the massaging tool, therefore through a predominant proactive mode of action control, where operations of forward and inverse modeling do not require sensory feedback. While these opposite modes of action do not seem to offer any compromise, we then discuss whether technological advances and collaborative robots might reconcile proactive and retroactive modes of action control during a massage, and offer new massage perspectives through a stochastic sensorimotor user experience. This transition faculty, from one mode of control to the other, might definitely represent an innovative conceptual approach in terms of human-machine interactions.

## Introduction

In the last decades, work and recreational activities drastically affected our habits by increasing sedentary life (Choi, 2019). This is further a well-admitted harmful consequence of the overuse of computers and smartphones resulting in various disorders such as postural deformity, and neck and shoulder pain (Choi, 2019). It is also well-established that the repetition and prolonged maintenance of body postures affects health (Kett and Sichting, 2020). To prevent and address such disorders, manual massage (MM) techniques are appreciated and recommended. However, these have not yet been considered as therapeutic interventions *per se*. Practically, MM target soft tissues such as muscles, tendons, and fascias (Guimberteau, 2004; Guimberteau et al., 2016). The MM treatment applied to these structures is performed through several maneuvers including effleurage, kneading, static or slide pressures, but also light and deep pressures. They are likely to involve biomechanical, physiological, neurological and psychological mechanisms, although these empirical attributions are not always confirmed by scientific data (Weerapong et al., 2005). While the benefits of MM are extensively demonstrated in the literature in individuals with or without motor disorders (Poppendieck et al., 2016), such interventions remain expensive and time-consuming (Weerapong et al., 2005). Delivering a MM requires a trained practitioner, and massaging interventions are difficult to scale since only one patient can be treated at the same time. To address these limitations, self-massage using foam-rolling (FR) became popular quite recently, especially in the sport domain. FR has applications in strength and conditioning, and is most frequently administered during warm-up and recovery. FR consists in making back and forth movements with a foam roller or a roller massager. FR thus consists in rolling actions applying pressure to soft tissues (Cheatham et al., 2015; Wiewelhove et al., 2019). Although direct comparisons between FR and MM in randomized control trials are limited (Sharp, 2012; Cho et al., 2015; Patole et al., 2019; Rivera et al., 2019), FR appears as conducive as MM to significantly improve performance (e.g., Schroeder and Best, 2015; Murray et al., 2016). Enhanced flexibility was observed in both athletes and non-athletes populations (Halperin et al., 2014; Kelly and Beardsley, 2016), and lower muscle pain was also reported after FR (Jay et al., 2014; Chan et al., 2015; Ceca et al., 2017). This accounted for an analgesic effect of FR, comparable to that found as a result of traditional MM (Monteiro et al., 2017b). Massaging techniques affect central pain-modulating sensory systems (nociceptors and mechanoreceptor sensitivity) (Capobianco et al., 2019). Also, mechanical pressure may alleviate pain perception through stimulation of afferent central nociceptive pathways and descending anti-nociceptive pathways, i.e., diffuse noxious inhibitory control (Wiewelhove et al., 2019). Both MM and FR might improve blood circulation and promote muscle homeostasis (i.e., rearrangements of myofascia, muscle fibers, and microvessels), although this claim awaits further experimental investigation (Schleip, 2003a, b).

As for MM, evidence for the benefits of FR remains foremost empirical. In spite of accumulating research, in practice, the physiotherapist can modify the parameters according to the people based on his own feelings and experience to achieve the expected result. The physiotherapist can also benefit in real-time from feedback from his patient during the massage intervention. FR being a self-administration, each practitioner retains some degrees of adjustments of the different parameters, as found with MM. These two configurations make it possible to individualize the treatment but does not facilitate the establishment of practical guidelines. The purpose of this review was to highlight the similarities in terms of scientific research (e.g., population, uses, outcome measures and effects) on MM and FR, and extend the discussion in the field of robotic massage. Currently, there is no literature review reporting the effects of these three massage modalities. The present paper first provides a synthesis of the experimental evidence supporting their benefits, and then disentangles optimal inherent characteristics and practical guidelines for efficient interventions. We also conceptualize the sensorimotor experience involved by MM and FR to delineate perspectives and research avenues. More specifically, we discuss whether MM should be considered a retroactive closed-loop convergence process while FR should rather be considered a proactive mode of action control. We consider to which extent future developments in the field, such as robotic solutions, might allow switching from a proactive to a retroactive mode of action control in specific massaging routines. We examine whether assisting classical massages with interactive and intelligent massaging robots might represent a promising and fruitful avenue.

This literature review was conducted using four search engines, Google Scholar, PubMeb, ResearchGate and Kinedoc, preceding and including 2020, without language restrictions. A total of 413 articles including key-words “foam roller,” “roller massager,” foam rolling,” “self-myofascial release,” “self-massage,” “MM,” “automated massage,” and “robotic massage,” were found in English, French and Spanish. A total of 316 articles were included. Items including the use of a foam roller or other comparative tool that did not include rolling the device on soft tissue were excluded (i.e., use for core stability training). A total of 173 articles were finally retained.

## Manual Massage

MM therapy is widely used as a warm-up method and cool-down process in sports (Weerapong et al., 2005). It is also administered in clinical populations for therapeutic purposes or well-being (Field et al., 2005). In the first case, therapeutic massage is practiced as part of therapeutic interventions targeting the symptoms of a specific pathology/disease. MM appears to be an effective treatment for infants of depressed mothers, and in elderly patients with severe dementia (Field et al., 1996; Suzuki et al., 2010). Also, world-class athletes benefit from MM to improve their performance and facilitate recovery (Espí-López et al., 2020). Noteworthy, the use of MM is not restricted to therapeutic or performance-enhancing interventions. Indeed, it is also used as a tool to promote well-being. Wellness massage is therefore not intended to treat patient, but also used for the sole purpose of enhancing perceived well-being across physical, mental, social and even spiritual domains (Andrade, 2013). The various maneuvers and pressures are performed on soft tissues by the hands of a qualified physiotherapist, who adjusts his MM routine based on the aim and time available for his intervention.

### Dependent Variables

#### Psychometric and Behavioral Assessments

MM is an ancestral practice found in many civilizations. Beyond these origins, it is available in three forms: wellness, therapeutic, and sports MM. To appreciate its impact, researchers first collected subjective evaluations through self-report ratings. These tools are simple to use, cost-effective, and non-invasive. While monitoring objective data is likely to provide greater levels of precision, it remains costly and invasive. There are nonetheless several reports of behavioral assessments of the effects of MMs. Joint amplitude is assessed by means of goniometers and functional tests (Leivadi et al., 1999; Hilbert et al., 2003; Zainuddin et al., 2005; McKechnie et al., 2007; Arabaci, 2008; Arazi et al., 2012; Iwamoto et al., 2016; Table 1). Behavioral measures also enabled researchers to assess the impact of MM on strength production of athletes (Rinder and Sutherland, 1995; Tiidus and Shoemaker, 1995; Farr et al., 2002; Hilbert et al., 2003; Dawson et al., 2004; Zainuddin et al., 2005; Jakeman et al., 2010). The influence of MM was also examined in vertical and horizontal power production (Farr et al., 2002; McKechnie et al., 2007; Willems et al., 2009; Jakeman et al., 2010; Delextrat et al., 2013; Abrantes et al., 2019). Speed and agility qualities were checked, taking into account acceleration, deceleration (Mancinelli et al., 2006; Arabaci, 2008; Arazi et al., 2012; Delextrat et al., 2013; Table 1).

**TABLE 1:** The effects of MM on performances.

Author (year)	Study design	Sample	Massage intervention			Control	Others experimental groups	Outcome measures	Effects
			Targeted area	Technique	Treatment time				
**Range of motion**									
Leivadi et al. (1999)	RCT	30 dance students	Whole body	Effleurage, petrissage, friction	30 min	No	Massage Relaxation therapy 2 times/week over 5 weeks	Neck and shoulder ROM	Long term effect: ↑ neck extension ROM ↑ shoulder abduction ROM
Arazi et al. (2012)	RCT	20 college athletes	Main lower limb muscles	Effleurage, friction, petrissage, vibration and tapotment	15 min	Yes	Swiss massage Static-stretching	Sit-and-reach	↑ lower back and hamstrings flexibility
Hilbert et al. (2003)	RCT	18 young and healthy subjects	Hamstrings	Effleurage, tapotement, petrissage	20 min/post 2 h	Yes	Swedish massage Placebo massage	Straight leg raise test	NS change hamstring ROM
McKechnie et al. (2007)	RC	19 healthy and recreationaly active	Plantar flexors	Effleurage and petrissage	3 min/leg	Yes	Petrissage Tapotementat at 4 Hz	Ankle joint flexibility	petrissage > tapotement ↑ ankle ROM
Arabaci (2008)	RC	24 healthy and physically active	Main lower limb muscles	Effleurage, friction, petrissage, vibration, tapotment	15 min	Yes	Swedish massage Stretching	Sit-and-reach	↑ lower back and hamstrings flexibility
Iwamoto et al. (2016)	CT	12 healthy students	Popliteal fossa	Small circle with thumb at 3 Hz	2–3 min	No	Friction massage	Ankle joint flexibility	↑ dorsiflexion NS change plantar flexion
**Power performance**									
Mancinelli et al. (2006)	RCT	22 NCAA Division I basketball and volleyball players	Quadriceps, hamstrings	Effleurage, petrissage and vibration	17 min/post 48 h	Yes	Western massage	CMJ Timed shuttle run	↑ vertical power NS change horizontal power
Arazi et al. (2012)	RCT	20 college athletes	Main lower limb muscles	Effleurage, friction, petrissage, vibration and tapotment	15 min	Yes	Swiss massage Static-stretching	Vertical jump, 30 m sprint, agility-T test	↓ horizontal and vertical power ↓ agility
Delextrat et al. (2013)	RCT	8 bsketball players	Main lower limb muscles and back	Effleurage, petrissage	15 min/leg	Yes	Massage Cold-water immersion	CMJ Repeated sprint ability	↑ CMJ NS change repeated sprint ability
Farr et al. (2002)	RCT	8 healthy and recreationnaly active	Main lower limb muscles	Effleurage, petrissage (no deep tissue massage)	30 min/post 2 h	No	Leg massage Leg control	Single limb jumps	↓ muscular power at 24 h
McKechnie et al. (2007)	RC	19 healthy and recreationaly active	Plantar flexors	Effleurage and pettrisage	3 min/leg	Yes	Petrissage Tapotementat at 4 Hz	Drop-jump	NS change muscular power
Arabaci (2008)	RC	24 healthy and physically active	Main lower limb muscles	Effleurage, friction, petrissage, vibration, tapotment	15 min	Yes	Swedish massage Stretching	30 m sprint Leg reaction time	↓ horizontal and vertical power ↓ reaction time
Jakeman et al. (2010)	RCT	32 healthy and physically active	Main lower limb muscles	Effleurage, petrissage, tapotment and hacking	30 min	Yes	Sport massage + compression Compression alone (12 h)	CMJ Squat jump (SJ)	↑ horizontal and vertical power
Abrantes et al. (2019)	RCT	39 physically active men	Finger/elbow Stick massage	Main lower limb muscles and chest	8 min	No	Manual massage (upper body) + Foam rolling (lower limbs)	Vertical and horizontal jump	↑ vertical and horizontal power
**Strength performance**									
Rinder and Sutherland (1995)	RC	20 club member	Quadriceps	Effleurage and petrissage	3 min/leg	Yes	Massage	Maximum number of leg extension at 50% 1RM	↑ quadriceps performance
Tiidus and Shoemaker (1995)	RCT	9 healthy students	Quadriceps	Superficial and deep effleurage	10 min/post < 1 h	No	Leg massage Leg control	Isometric and isokinetic knee extension	NS change muscle strength
Zainuddin et al. (2005)	RC	10 healthy subjects	Hand and main upper limb muscles	Effleurage, petrissage, friction	10 min/post 3 h	No	Arm massage Arm control	Isometric and isokinetic elbow flexion	NS change muscle strength
Willems et al. (2009)	RCT	7 healthy and moderately active	Main lower limb muscles	Effleurage, petrissage, tapotement	25 min	No	Leg massage Leg control	Single limb jumps	↑ muscular power at 48 h
Jakeman et al. (2010)	RCT	32 healthy and physically active	Main lower limb muscles	Effleurage, petrissage, tapotment and hacking	30 min	Yes	Sport massage + compression Compression alone (12 h)	Knee extension	↑ isokinetic strength vs control group
Abrantes et al. (2019)	RCT	39 physically active men	Finger/elbow Stick massage	Main lower limb muscles and chest	8 min	No	Manual massage (upper body) + Foam rolling (lower limbs)	Vertical and horizontal jump	↑ vertical and horizontal power

*CT, Clinical trial; RCT, randomized controlled trial; RC, randomized crossover; CCT, controlled clinical trial; NR, not reported; ROM, range of motion; CMJ, countermovement jump; ↑ indicates increase; ↓ indicates decrease; NS, not significant.*

#### Neurophysiological Evaluations

Technological advances allowed investigation of changes occurring at the physiological level (Table 2). For instance, cutaneous temperature attesting changes in peripheral blood circulation was frequently collected (Drust et al., 2003; Hinds et al., 2004; Mori et al., 2004). It thus became possible to evaluate muscle temperature (Drust et al., 2003; Hinds et al., 2004), speed of blood circulation (Tiidus and Shoemaker, 1995; Hinds et al., 2004; Mori et al., 2004; Wiltshire et al., 2010), and blood pressure (Hinds et al., 2004; Arroyo-Morales et al., 2008; Wiltshire et al., 2010; Table 2). Some researchers also investigated the repercussions of MM on the activation of the sympathetic and parasympathetic nervous systems through monitoring of heart rate variability (Hemmings et al., 2000; Drust et al., 2003; Robertson et al., 2004; Arroyo-Morales et al., 2008; Wiltshire et al., 2010; Pinar et al., 2012; Table 2). More invasive procedures, such as biopsies and blood sampling, enable measures of changes in cortisol levels, markers of inflammation and metabolic products (Kaada and Torsteinb, 1989; Smith et al., 1994; Leivadi et al., 1999; Hemmings et al., 2000; Hilbert et al., 2003; Hinds et al., 2004; Robertson et al., 2004; Zainuddin et al., 2005; Ogai et al., 2008; Cupido, 2010; Rapaport et al., 2010; Wiltshire et al., 2010; Crane et al., 2012; Pinar et al., 2012; Iwamoto et al., 2016; Kargarfard et al., 2016; White et al., 2020; Table 2). This represents valuable information to prevent stress or inflammatory conditions that may ultimately lead to injury or a state of overtraining.

**TABLE 2:** The effects of MM on neurophysiological and psychological variables.

Author (year)	Study design	Sample	Massage intervention			Control	Others experimental groups	Outcome measures	Effects
			Targeted area	Technique	Treatment time and rate				
**Neurophysiological and physiological effects**									
Leivadi et al. (1999)	RCT	30 dance students	Whole body	Effleurage, petrissage, friction	30 min/Slow	No	Massage Relaxation therapy 2 times/week over 5-weeks	Salivary cortisol	↓ cortisol (stress hormones)
Hemmings et al. (2000)	RCT	8 amateur boxers	Main lower limb muscles, back, shoulder and arms	Effleurage, petrissage	20 min Effleurage: 30 strokes/min Petrissage: 50–60 strokes/min	Yes	Massage therapy	Blood analyzes Heart rate	NS change blood lactate, glucose concentration NS change heart rate
Kargarfard et al. (2016)	RCT	30 male bodybuilders	Quadriceps	Effleurage, petrissage and vibration	30 min/post 2 h	Yes	Western massage group	Blood sample	Massage vs control group : ↓ creatine kinase level from 48 h up to 72 h
White et al. (2020)	RC	9 collegiate-level athletes	Main lower limb muscles	Effleurage and neurolymphatic	30 min	Yes	Massage therapy	Blood sample pre- and post-exercise T0, T+1, 2 h, 24 h	↓ inflammation marker concentrations (↓IL-6)
Kaada and Torsteinb (1989)	CT	12 subjects with chronic pain	Lumbo-sacral region	Connective tissue massage	30 min/Slow	No	Connective tissue massage	Blood sample	↑ relaxation substances (↑ ß-endorphins)
Smith et al. (1994)	RCT	14 healthy but untrained subjects	Biceps, triceps	Effleurage, shaking, petrissage, cross-fibre	30 min/post 2 h	Yes	Sport massage Sham massage	Blood analyzes	↑ neutrophils ↓ CK Less ↓ cortisol serum
Tiidus and Shoemaker (1995)	RCT	9 healthy students	Quadriceps	Superficial and deep effleurage	10 min/post < 1 h	No	Leg massage Leg control	Arterial blood velocity Venous blood velocity	NS change quadriceps muscle blood flow
Drust et al. (2003)	RC	7 healthy subjects	Quadriceps	Deep effleurage	5, 10, 15 min 52 strokes/min	No	3 groups of massage Ultrasound (5 min)	Heart rate monitor Skin and intramuscular temperature	↑ intra muscular and skin temperature at depths of 1.5 and 2.5 cm ↑ heart rate
Hilbert et al. (2003)	RCT	18 young and healthy subjects	Hamstrings	Effleurage, tapotement, petrissage	20 min/post 2 h	Yes	Swedish massage Placebo massage	Blood sample	NS change neutrophils
Hinds et al. (2004)	RCT	13 yound healthy subjects	Quadriceps	Deep effleurage and petrissage	2*6 min 50–60 strokes/min	Yes	Massage	Blood flow Skin and muscle temperature Blood sample Blood pressure Heart rate	↑ skin temperature ↑ skin blood flow NS change blood presusre, heart rate, lactate concentration and FABF
Robertson et al. (2004)	RC	9 healthy and recreationnaly active	Main lower limb muscles	Effleurage, kneading, picking up, wringing, rolling	20 min/post active recovery 5 min	Yes	Massage	Blood sample Heart rate	NS change lactate level and heart rate response
Zainuddin et al. (2005)	RC	10 healthy subjects	Hand and main upper limb muscles	Effleurage, petrissage, friction	10 min/post 3 h	No	Arm massage Arm control	Upper arm circumference Blood sample	↓ swelling smaller ↑ creatine kinase activity
Arroyo-Morales et al. (2008)	RCT	62 healthy active subjects	Whole-body myofascial release	Long stroke, cross hand, static pressure	40 min/post 15 min active and passive recovery	Yes	Sham (ultrasound and magnetotherapy)	Heart rate variability Bood pressure	↓ heart rate variability index ↓ diastolic blood pressure
Ogai et al. (2008)	RC	11 healthy and active students	Main lower limb muscles	Petrissage	10 min between two sets	Yes	Massage	Blood sample	NS blood lactate concentration
Cupido (2010)	RC	13 young healthy and recreationnaly active	Quadriceps	Effleurage, petrissage, compression	10 min/post 10 min	No	Leg massage Leg control	Blood sample Muscle damage Muscle glucose level	NS glycogen, lactate concentration and muscle damage
Jakeman et al. (2010)	RCT	32 healthy and physically active	Main lower limb muscles	Effleurage, petrissage, tapotment and hacking	30 min	Yes	Sport massage + compression Compression alone (12 h)	Blood sample	NS creatine kinase activity
Rapaport et al. (2010)	RCT	53 healthy subjects	Full body	Effleurage, petrissage, kneading, tapotement and thumb friction	45 min	No	Swedish massage Light touch	Blood analyzes T+5, +1 pre- min and post- T+1, 5, 10, 15, 30, 60 min	Massage > light touch ↑ immune system (↑ circulating phenotic lymphocyte, ↓ cytokine level, arginine-vasopressin and cortisol)
Wiltshire et al. (2010)	RC	12 healthy subjects	Forearm muscles	Effleurage, petrissage	10 min	Yes	Massage Active recovery	Forearm blood flow Blood sample Heart rate	↓ blood flow (impairing lactic acid removal) ↓ heart rate vs active recovery NS change heart rate vs passive recovery
Crane et al. (2012)	RCT	11 young healthy and recreationnaly active	Quadriceps	Effleurage, petrissage, compression	10 min/post 10 min	No	Leg massage Leg control	Blood analyzes	↓ inflammation (cytokines TNF-a, interleukin-6, heat shock protein 27) ↑ mitochondrial biogenesis (focal adhesion kinase, ERK1/2, PGC-1a) NS change muscle metabolites (glyocgen, lactate)
Pinar et al. (2012)	RC	12 young healthy and recreationnaly active	Quadriceps, hamstrings	Effleurage, kneading, picking up, wringing, rolling	24 min	Yes	Massage Electrical muscle stimulation	Blood sample Heart rate	NS blood lactate concentration and heart rate
Iwamoto et al. (2016)	CT	12 healthy students	Popliteal fossa	Small circle with thumb at 3 Hz	2–3 min	No	Friction massage	Oxygenated hemoglobin Deoxygenated hemoglobin Total hemoglobin	↑ venous return (muscle oxygenation)
**Psychological effect**									
Leivadi et al. (1999)	RCT	30 dance students	Whole body	Effleurage, petrissage, friction	30 min/slow	No	Massage Relaxation therapy 2 times/week over 5 weeks	State-trait anxiety inventory Profil of mood states Pain VAS-10	↑ mood ↓ anxiety ↓ pain
Hemmings et al. (2000)	RCT	8 amateur boxers	Main lower limb muscles, back, shoulder and arms	Effleurage, petrissage	20 min 30–60 strokes/min	Yes	Massage	Numerical recovery scale	↑ perceived recovery
Carcano et al. (2010)	CT	96 national and international atheletes	Main lower limb muscles	Superifical and deep effleurage, friction	20–30 min/Slow	No	Swedish massage	Pain VAS-10 Fatigue VAS-10 Well being VAS-10	↓ muscle soreness ↓ muscular fatigue ↑ well-being
Delextrat et al. (2013)	RCT	8 bsketball players	Main lower limb muscles and back	Effleurage, petrissage	15 min/leg	Yes	Massage Cold-water immersion	Overall fatigue VAS-10	↓ perceived fatigue
Jourdain (2015)	CT	11 young athletes	Main lower limb muscles	Longitudinal/transverse deep sliding pressures, kneading and circular friction	10 min/leg post 2 h	No	Massage 1 time/week over 5-weeks	HPHEES Scale	↓ perceived fatigue on waking NS change overall physical form
Visconti et al. (2015)	Pilot study	25 ultramarathon runners	Main lower limb muscles	Effleurage	20 min	No	Massage	Numeric pain rating scale Patient global impression of change	↓ muscle pain
Kargarfard et al. (2016)	RCT	30 male bodybuilders	Quadriceps	Effleurage, petrissage and vibration	30 min/post 2 h	Yes	Western massage group	Pain VAS-10	↓ muscle soreness from 24 h up to 72 h
Mori et al. (2004)	RC	29 healthy students	Lumbar and sacrum region	Effleurage, kneading and compression techniques	5 min between two sets	Yes	Massage	Fatigue VAS-10	↓ perceived fatigue
Robertson et al. (2004)	RC	9 healthy and recreationnaly active	Main lower limb muscles	Effleurage, kneading, picking up, wringing, rolling	20 min/post-active recovery 5 min	Yes	Massage	Fatigue index	↓ perceived fatigue
Sharpe et al. (2007)	RCT	54 elderly subjects (≥ 60 years)	Whole body	Swedish, neuromuscular, and myofascial techniques	50 min	No	Massage therapy Guided relaxation 2 times/week over 4-weeks	General well-being schedule Perceived stress scale	↓ anxiety, depression ↑ vitality, general health and positive well-being vs guided relaxation group
Ogai et al. (2008)	RC	11 healthy and active students	Main lower limb muscles	Petrissage	10 min between two sets	Yes	Massage	Perceived fatigue VAS-10	↑ perceived recovery between two high intensive exercises
Pinar et al. (2012)	RC	12 young healthy and recreationnaly active	Quadriceps, hamstrings	Effleurage, kneading, picking up, wringing, rolling	24 min	Yes	Massage Electrical muscle stimulation	Total quality of recoveryRating of perceived exertion	NS change psychological recovery after high intensity exercise
Nakano et al. (2019)	RC	12 elderly people (65 years old)	HandsFeet	Stroke	15 min	No	Hand massage Foot massage	Likert scale	Both groups ↑ pleasant, relaxed and refreshed feelings

*CT, clinical trial; RCT, randomized controlled trial; RC, randomized crossover; VAS, visual analogue scale; NR, not reported; ↑ indicates increase; ↓ indicates decrease; NS, not significant.*

#### Short-Term Effects

MM therapy has supposedly many virtues (Calvert, 2002). Its positive effects have been extensively reported in the scientific literature (Weerapong et al., 2005; Best et al., 2008; Brummitt, 2008). A sensation of psychological well-being was frequently reported (Mancinelli et al., 2006; Visconti et al., 2015). MM is further supposed to alleviate mood and anxiety disorders (Leivadi et al., 1999; Sharpe et al., 2007; Nakano et al., 2019), improve the feeling of recovery, reduce physical fatigue (Hemmings et al., 2000; Mori et al., 2004; Robertson et al., 2004; Ogai et al., 2008; Carcano et al., 2010; Pinar et al., 2012; Delextrat et al., 2013; Jourdain, 2015).

Several authors reported range of motion (ROM) gains. Indeed, the dorsiflexion following MM ranged from 18.4° to 22.8°. Also, on a sit-and-reach box test, participants’ score increased from 11.8 to 12.7 cm, after only 15 min of MM (Arabaci, 2008; Iwamoto et al., 2016). More generally, MM were shown to induce short-term flexibility gains, similar to those induced by static stretching, without co-occurrence of negative effects on physical performance (McKechnie et al., 2007; Arazi et al., 2012). These notions of ROM and flexibility were combined to increase the suppleness of an athlete.

In terms of recovery, few studies measured the influence of manual therapy on muscle stiffness. To date, no consensus is clearly established. Authors observed, using a durometer, a drop in stiffness between two intense efforts after a kneading MM (Ogai et al., 2008). Ultrasound shear wave elastography also showed progress in stiffness, but the benefits did not last more than 3 min (Eriksson Crommert et al., 2015). Some contradictory results, obtained with a rotary potentiometer or a myotonometry device, could be due to the very short observation time (Thomson et al., 2015; Kong et al., 2018). Before drawing general conclusions, further experimental studies are certainly required. Particular attention should be paid to the timing of the measurements. Manual therapy further appears to be effective to reduce adverse effects of exercise, likely to elicit delayed-onset muscle damage (DOMS). These benefits were obtained when MM is performed immediately after the effort, and up to 3 h afterward; Table 3). A recent meta-analysis concluded that MM could be the most efficient post-exercise intervention to prompt recovery. Compared to cryotherapy, cold-water immersion and compression garment, MM elicited a greater reduction in DOMS, perceived fatigue, and markers of inflammation (Dupuy et al., 2018). With regards to sport performance, MM was punctually found to positively affect the recovery of muscle power (Mancinelli et al., 2006; Willems et al., 2009) Table 1). However, other studies failed to detect such positive changes (Tiidus and Shoemaker, 1995; Farr et al., 2002), while others reported negative effects (Arabaci, 2008; Arazi et al., 2012). Similar inconsistent results were also reported by various meta-analytical reviews (Brummitt, 2008; Gaullier, 2015; Poppendieck et al., 2016). In another set of studies, MM was not found to promote force reduction after an exercise-induced muscle damage (Tiidus and Shoemaker, 1995; Farr et al., 2002; Hilbert et al., 2003; Zainuddin et al., 2005; Table 3). Based on these data, and despite some inconsistencies, we shall recommend the use of MM before a physical effort or even between two successive sporting events. Also, athletes immobilized due to injury could benefit from MM. In mice, Saitou et al. (2018) demonstrated that mechanical interventions mimicking MM could modulate inflammatory responses by local effects on interstitial fluid dynamics. The pressure exerted would induce a shear stress exertion on macrophages in situ, attenuating the phenomenon of muscle atrophy by a lymphatic and immune response (Saitou et al., 2018; Sakitani et al., 2019). In animal models, MM induces numerous neurophysiological changes. In fact, MM was associated with modulations in neural, lymphatic, and genetic responses (Lima et al., 2020). For example, abdominal massage improves transit in rats, i.e., reduced time to first fecal discharge in response to mechanical pressures. At the endocrine level, it was also shown that MM reduced the levels of gastrointestinal hormones, i.e., insulin, gastrin and somatostatin (Lima et al., 2020). MM also had modulatory effects at the neural level, since its analgesic effects were associated with changes in descending pain modulation circuits (Vigotsky and Bruhns, 2015). Nonetheless, the lack of consistency in the experimental findings in humans might be explained by a weak methodological rigor, as few protocols were reproduced and tested, hence supporting that there is no clear and precise design ensuring effectiveness of the intervention. The high variability of the studies is well-illustrated by the use of effleurage and petrissage techniques, while others also used friction, picking up, and shaking techniques. Likewise, the number of areas treated and the effective time of sport MM could fluctuate from 5 to 30 min (Brummitt, 2008; Poppendieck et al., 2016). Similarly, very few studies specified the intensity, the speed and the gestural frequency exerted by the therapist during the MM. Although these data are difficult to quantify, these parameters remain essential, as is the experience of the therapist, which has a main influence on MM effectiveness, and should be more rigorously controlled. Accordingly, Moraska (2007) provided evidence that therapist with 950 h of didactic training achieved significantly better results in muscle soreness than with 450 or 700 h of training. Although MM is an ancestral practice, this therapy, which is above all empirical, retains a certain number of gray areas, particularly in terms of sports massage, and the standardization of a sport MM protocol is warranted.

**TABLE 3:** The effects of MM on delayed-onset muscle soreness.

Author (year)	Study design	Sample	Massage intervention			Nature of the exercise	Control	Others experimental groups	Outcome measures	Effects
			Targeted area	Technique	Treatment time					
Mancinelli et al. (2006)	RCT	22 NCAA Division I basketball and volleyball players	Thigh	Effleurage, petrissage, vibration	17 min/post 48 h	Intense strength training and drills	Yes	Western massage	PPT in quadriceps femoris Muscle soreness VAS-10 Vertical jump	↓ DOMS ↑ tenderness ↑ vertical power
Kargarfard et al. (2018)	RCT	30 male bodybuilders	Quadriceps	Effleurage, petrissage and vibratioon	30 min/post 2 h	5 sets of squat until exhaustion at 75% of 1-RM	Yes	Western massage	Pain VAS-10 CMJ Blood sample Isometric torque pre- and post- T0, T+24, 48 and 72 h	Massage ↓ muscle soreness at 24, 48 and 72 h ↓ creatine kinase from 48 h ↑ vertical power and muscle strength at 48 h
Smith et al. (1994)	RCT	14 healthy but untrained subjects	Biceps, triceps	Effleurage, shaking, petrissage, cross-fibre	30 min/post 2 h	Biceps and triceps eccentric exercise	Yes	Sport massage Sham massage	Clarkson Scale Blood creatine kinase concentration pre- and post- T0, T+8, 24, 48, 72, 96, 120 h Bood analyses (neutrophils and cortisol) pre- and post- T0, T+8 h (30-minute intervals)	↓ DOMS intensity Peak DOMS at 24 h ↓ markers damage and inflammation (creatine kinase, cortisol) ↑ neutrophils activity
Tiidus and Shoemaker (1995)	CCT	9 healthy students	Quadriceps	Superficial and deep effleurage	10 min/post < 1 h	Quadriceps eccentric exercises	No	Leg massage Leg control	Numerical pain-rating scale Isometric and isokinetic knee extension pre- and post- T+15 min, T+24, 48, 72, 96 h	Tendency ↓ perception of DOMS from 48 h Peak DOMS at 24 h NS change muscle strength
Farr et al. (2002)	RCT	8 healthy and recreationnaly active	Main lower limb muscles	Effleurage, petrissage (no deep tissue massage)	30 min/post 2 h	40 min downhill treadmill walk loaded	No	Leg massage Leg control	Clarkson Scale PPT Isometric and isokinetic knee extension Vertical jump pre- and post- T0, T+24, 48, 72, 96, 120 h	Tendency ↓ DOMS magnitude ↓ muscle tenderness attenuate the decrease of strength and vertical power
Hilbert et al. (2003)	RCT	18 young and healthy subjects	Hamstrings	Effleurage, tapotement, petrissage	20 min/post 2 h	Hamstrings eccentric exercises	Yes	Swedish massage Placebo massage	Differential descriptor scale intensity of soreness Blood sample Eccentric hamstring contraction pre- and post- T0, T+2, 6, 24, 48 h	↓ perception of DOMS from 48 h Peak DOMS at 24 h NS change neutrophils, ROM and peak torque
Zainuddin et al. (2005)	RC	10 healthy subjects	Hand and main upper limb muscles	Effleurage, petrissage, friction	10 min/post 3 h	Elbow flexors eccentric exercises	No	Arm massage Arm control	Pain VAS-10 Isometric and isokinetic elbow flexor strength Blood sample pre- and post- T0, T+1, 2, 3, 4, 7, 10, 14 days	↓ DOMS magnitude for palaption and joint mobilization ↓ creatine kinase activity NS change muscle strength
Willems et al. (2009)	RCT	7 healthy and moderately active	Quadriceps	Effleurage, petrissage	25 min	20 min downhill treadmill walking at 25% decline	No	Leg massage Leg control	Quadriceps pain VAS-10 Single limb jumps pre- and post- T+24, 48, 72 h	↓ DOMS vastus lateralis and rectus femoris at 48 h ↑ vertical power at 48 h

*CT, clinical trial; RCT, randomized controlled trial; RC, randomized crossover; CCT, controlled clinical trial; DOMS, delayed onset muscle soreness; NR, not reported; ↑ indicates increase; ↓ indicates decrease; NS, not significant.*

#### Long-Term Intervention

To prepare the body for an intense exercise or to facilitate the post-exercise recovery, longer-term repeated MM interventions have extensively been administered within a span of 2–5 weeks. One to two weekly MM sessions of 20–30 min were found to reduce the level of stress and fatigue (Leivadi et al., 1999; Jourdain, 2015). After a long-term exercise, a difference was noticed by the athletes after receiving a one-sided manual leg massage. According to the participants, the perception of recovery of the side massaged was greater than the control (Dawson et al., 2004). With regards to well-being, it seems that a regular MM makes increase neck and shoulder ROM (Leivadi et al., 1999; Yang et al., 2012). MM finally contributed to decrease the level of salivary cortisol after a period of 5 weeks, after a design including two massages per week (Leivadi et al., 1999). These various effects concurred with increased relaxation resulting from the activation of the parasympathetic nervous system.

### Experimental Procedures

MM is universally appreciated. Classical guidelines emphasized the importance of dynamic movements for stimulating the soft tissues (e.g., vibration and tapotement). Slow gestures (e.g., effleurage, kneading, sliding pressure and friction) were recommended for well-being and relaxation. When the MM is designed to promote post-exercise recovery, effleurage and kneading should rather be preferred (Standley et al., 2010). Incorporating tapotements was further relevant to reduce DOMS and joint amplitude recovery, whereas vibration facilitated blood circulation and friction promoted relaxation (Standley et al., 2010). A cool-down MM routine using might last between 15 and 30 min to allow physiological changes (Standley et al., 2010). Despite guidelines, future experimental designs should consider and study the pressure levels and the gestural speed of the practitioner during MM to compensate for the lack of data.

## Foam Rolling

FR is a self-myofascial release technique requiring direct contact with the skin, where fingers or tools are used to slowly press the fascial tissue. FR have extensively been adopted in fitness and conditioning communities in recent years (Cheatham and Stull, 2018a, b). Because of its simplicity and measurable effects, FR is administered as part of warm-up and recovery routines (Fleckenstein et al., 2017). Practically, FR administered using a foam roller, a roller massager, sticks or balls with varying sizes and density, further became very popular to improve functional outcomes such as ROM and pain pressure threshold (PPT). The first technique consists in performing simple back and forth movements, thus exerting mechanical pressures on soft tissues via the weight of the body (or the force of the upper limbs). A second technique, called ischemic pressure, requires a static pressure during a 6–30 s period, below the individual pain tolerance threshold (Abels, 2013; Myers, 2013; Kalichman and Ben David, 2017). This complementary approach is designed to reduce pain felt and improve ROM, but requires greater expertise with FR (Kalichman and Ben David, 2017). For users, the goal is to get closer from MM practice of the physiotherapist, more specifically to reproduce the method of Rolfing.

**FIGURE 1:**
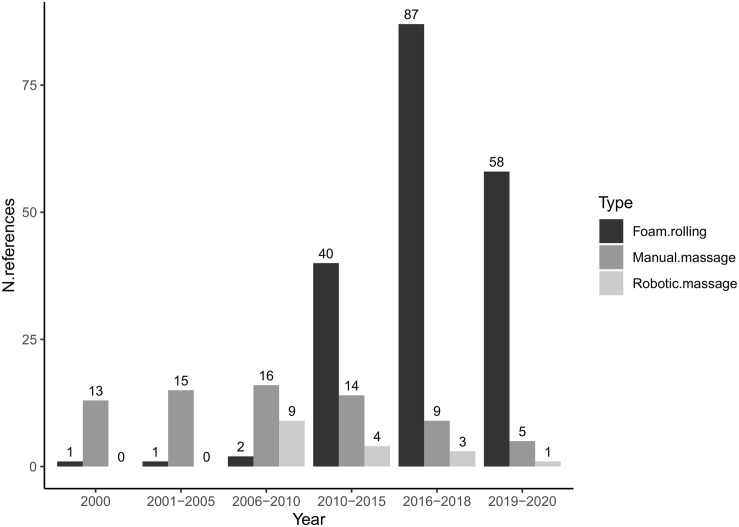
The evolution of scientific interest according to the type of massage.

Although the scientific literature addressing the effects of FR remains sparse, this research topic is currently gaining attention (Cheatham et al., 2015; Wiewelhove et al., 2019; Figure 1). There is an emerging consensus that FR positively affects athletic performances such as power, strength, agility, balance and flexibility (Schroeder and Best, 2015). FR also yielded beneficial effects in rehabilitation settings with elderly populations or patients suffering from locomotor disorder such as genu varus (Jafarnezhadgero et al., 2018; Lee and Lim, 2018), round shoulder posture (Choi, 2019), or spastic diplegia (Patole et al., 2019). In the same vein, recent FR studies investigated its potential effectiveness in the context of rehabilitation (i.e., tendinopathies, friction syndrome of the iliotibial band, fibromyalgia, myofascial pain syndrome, or postural correction) (Grieve et al., 2013; Aboodarda et al., 2015; Chan et al., 2015; Ceca et al., 2017; Lee et al., 2017; Jafarnezhadgero et al., 2018). The theoretical rationale advanced as an account to the benefits of FR largely overlaps that for traditional MM.

### Dependent Variables

#### Psychometric and Behavioral Assessments

As for MM, experimental designs seeking to assess the effectiveness of FR involved psychometric, behavioral and physiological measures. Subjective measures primarily consisted in standardized questionnaires and self-reports ratings on Likert-type scales to quantify pain and quality of life (Healey et al., 2014; Cavanaugh, 2016; Fleckenstein et al., 2017; Table 4). Researchers also used Borg scales or numerical ratings scale as means to collect perceived recovery (Healey et al., 2014; Peacock et al., 2015; Fleckenstein et al., 2017; Kalén et al., 2017; Rey et al., 2017; Beier et al., 2019; Table 4). Considering that pain is not objectively measurable, these different scales offer a simple solution to assess the quality and speed of recovery of DOMS (Jay et al., 2014; MacDonald et al., 2014; Romero-Moraleda et al., 2017, 2019; Naderi et al., 2019; Table 5). The algometer is also used to measure the PPT reliably, both at the muscular and joint levels, after FR routine. It is also used after exercise induced muscle damage and FR recovery (Pearcey et al., 2015; Correira, 2016; Casanova et al., 2017; Drinkwater et al., 2019; Table 5). Likewise, just like MM, functional tests, such as the sit-and-reach or weight-bearing lunge tests, are usual tools to assess the effectiveness of FR on ROM (Sullivan et al., 2013; Halperin et al., 2014; Grieve et al., 2015; Peacock et al., 2015; Škarabot et al., 2015; Kelly and Beardsley, 2016; Patel et al., 2016; Boguszewski et al., 2017; Grabow et al., 2017; Jung et al., 2017; Paz et al., 2017; Sağiroğlu, 2017; Garcia-Gutiérrez et al., 2018; Phillips et al., 2018; Guillot et al., 2019; Pathania and Muragod, 2019; Somers et al., 2019; Williams and Selkow, 2019; Table 6). Other clinical examinations, such as the Thomas test or the straight leg raise test, are also regularly used to quantify flexibility with manual or electric goniometers after FR (MacDonald et al., 2014; Mohr et al., 2014; Cho et al., 2015; Vigotsky et al., 2015; Su et al., 2017; Do et al., 2018; Killen et al., 2018; Madoni et al., 2018; Guillot et al., 2019; Jeong et al., 2019; Kyranoudis et al., 2019; Lim and Park, 2019; Oranchuk et al., 2019; Table 6). Applied to FR research, goniometer/inclinometer index gains in terms of degrees of freedom of the joints (Peacock et al., 2015; Škarabot et al., 2015; Fairall et al., 2017; Le Gal et al., 2018; Table 6). In addition to such flexibility tests, evaluating the effects of FR on physical qualities such as muscular power/strength, agility, and muscular activation, remains crucial (Mikesky et al., 2002; Fama and Bueti, 2011; Healey et al., 2014; Peacock et al., 2014, 2015).

**TABLE 4:** The effects of foam rolling on psychological variables.

Author (year)	Study design	Sample	Foam rolling intervention				Control	Others experimental groups	Outcome measures	Effects
			Tool(s)	Targeted area	Treatment time and rate	Intensity				
Peacock et al. (2015)	RC	16 athletically trained adult	High density FR	Main lower limb muscles and lower back	1 × 30 s/muscle10 bpm	NR	No	Sagittal plane FR Frontal plane FR	Borg scale	No change rate of perceived exhaustion
Kalén et al. (2017)	RC	12 surf lifeguards	High density FR	Main lower limb muscles per leg	2 × 60 s	7/10	Yes	Foam rolling Running Passive recovery	Borg CR-10 scale	No change rate of perceived exhaustion
Rey et al. (2017)	RCT	18 professional soccer players	High density FR	Main lower limb muscles	1 × 45 s/leg and muscle group 50 bpm	Much pressure as they could	Yes	Foam rolling	Total quality recovery scale Perceived muscle soreness VAS-7	↑ feelings of recovery ↓ perceived muscle soreness at 24 h post-training
Beier et al. (2019)	RC	11 resistance trained subjects	Stick massage	Rectus femoris and gluteus maximus	2 min/muscle	Heavy pressure	No	Foam rolling Dynamic warmup-up	Recovery scale-10	NS change perceived recovery
Healey et al. (2014)	RC	26 healthy college-aged and recreationally active	High density FR	Main lower limb muscles and upper back	1 × 30 s/muscles	Self-selected	Yes	Foam rolling Planking exercises	Borg CR-10 scale Fatigue VAS-10 Muscle soreness VAS-10 Palpating rating soreness scale	↓ fatigue NS difference muscle soreness
Cavanaugh (2016)	RC	12 healthy and recreationnaly active	Roller massager	Plantar flexors	3 × 30 s 15 bpm	7/100/10	Yes	Foam rolling ipsilaetral leg (7/10) Foam rolling contralateral leg (7/10) Sham (0/10)	Pain VAS-10	↓ pain perception with heavy foam rolling
Cheatham et al. (2017)	RCT	45 healthy subjects	High density FR	Quadriceps	1 × 120 s	Moderate	Yes	Video-guided Live-instructed Self-guided foam rolling + knee mobilizations	Quadriceps PPT	All groups : ↑ muscle tenderness
Cheatham et al. (2019)	RCT	45 healthy and recreationnaly	Vibrating foam roller Non vibrating foam roller	Quadriceps	1 × 120 s	Moderate	Yes	Vibratinh foam rolling (33 Hz) Non-ibrating foam rolling + knee mobilizations	Quadriceps PPT Passive knee flexion ROM	Both groups ↑ knee ROM ↑ muscle tenderness (vibrating > non-vibrating)
Cheatham and Baker (2017)	RCT	20 healthy subjects	High density FR	Quadriceps	1 × 120 s 1 inch per second	Moderate	No	Foam rolling leg Control leg + knee mobilizations	Quadriceps and hamstrings PPT	↑ muscle tenderness (crossover effect on contralateral quadriceps)
Fleckenstein et al. (2017)	RCT	55 healthy and recreationally active	High density FR	Mam lower limb muscles	1 × 30 s/muscle 60 bpm	7/10	Yes	Prevention Regeneration	Fatigue VAS-10 Pain VAS-10	↓ perceived fatigue (regeneration > prevention)
Han et al. (2017)	RCT	30 students patients will trigger point	Vibrating FR Non-vibrating FR	Mam lower limb muscles	1 × 20 min	NR	No	Vibrating FR (62 Hz) Non-vibrating FR 3 times/week over 4-weeks	Iiotibial-band, gluteus, rectus femoris, hamstrings PPT	↑ muscle tenderness in ITB Trend ↑ for others muscles
Cheatham and Stull (2018a)	RCT	21 healthy subjects	High density FR	Quadriceps	1 × 120 s 1 inch per second	Moderate	No	Foam rolling leg Control leg + knee mobilizations	Quadriceps and hamstrings PPT	↑ muscle tenderness (crossover effect on contralateral quadriceps)
Cheatham and Stull (2018a)	RCT	36 healthy and recreationally active	Soft FR Medium FR Hard FR	Quadriceps	1 × 120 s 1 inch per second	NR	No	Soft density FR Medium density FR Hard density FR + knee mobilizations	Quadriceps PPT	Three groups ↑ muscle tenderness
Cheatham and Stull (2018b)	RCT	30 healthy and recreationally active	High density FR	Quadriceps	1 × 120 s 1 inch per second	NR	No	Foam rolling only Foam rolling + knee mobilizations	Quadriceps PPT	↑ muscle tenderness (foam rolling with knee mobilizations > foam rolling only)
Cheatham et al. (2019)	RCT	45 healthy and recreationally active	Vibrating FR Non vibrating FR	Quadriceps	1 × 120 s 1 inch per second	NR	Yes	Vibrating FR(33 Hz) Non-vibrating FR Static stretching	Quadriceps PPT	↑ muscle tenderness (vibrating > non-vibrating FR)

*RC, randomized crossover; RCT, randomized controlled trial; FR: foam roller; NR, not reported; bpm, beats per minute: ↑ indicates increase; ↓ indicates decrease; VAS, visual analogue scale: PPT, pain pressure threshold; NS, not statistically significant.*

**TABLE 5:** The effects of foam rolling on delayed-onset muscle soreness.

Author (year)	Study design	Sample	Foam rolling intervention					Nature of exercise	Control	Others experimental groups	Outcome measures	Effects
			Tool(s)	Targeted area	Treatment time and rate	Intensity	Experience					
Casanova et al. (2017)	RC	10 athletes	Roller massager	Plantar flexors	6 × 45 s 30 bpm	NR	1 test session	5 × 201-leg calf raise at BW	Yes	Foam rolling leg Control leg	Gastrocnemius PPT Ankle dorsiflexion Muscle morphology Muscle oxygenation Plantar flexion, dorsiflexion MVIC pre- and post-T0, T+1, 24, 48, 72 h	↑ muscle tenderness ↑ ankle ROM NS change muscle oxygenation (HHb concentration) NS change muscle morphology NS change muscle performance
Jay et al. (2014)	RCT	22 healthy untrained	Roller massager	Hamstring	1 × 10 min 15–30 bpm	Moderate	NR	10 × 10 stiff-legged deadlift up to 32 kg	Yes	Foam rolling leg Control leg	Pain VAS-10 HamstringsPPT 1-leg sit-and-reach box test pre- and post- T0, T+10, 30, 60 min	↑ muscle tenderness up to 60 min ↓ muscle soreness up to 60 min ↑ ROM at 10 min Controlateral effect ↓ muscle soreness Tend ↑ muscle tenderness Trend ↑ ROM
MacDonald et al. (2014)	RCT	20 healthy and recreationally active	High density FR	Main lower limb muscles	2 × 60 s/muscle	NR	1 test session	10 × 10 squat at 60% of 1-RM	Yes	Foam rolling	BS-11 NRS Modified kneeling lunge Physical test and contractile properties pre- and post-T0, T+48, 72h	↓ muscle soreness ↑ performances ↑ muscle activity
Pearcey et al. (2015)	RC	8 healthy and physically active	High density FR	Main lower limb muscles	2 × 45 s/muscle 50 bpm	Much pressure as they could	Yes	10 × 10 squat at 60% of 1-RM	Yes	Foam rolling	Quadriceps PPT Physical tests pre- and post-T+24, 48, 72 h	↑ muscle tenderness ↑ physical performance decrements
Correira (2016)	RC	10 healthy and recreationnaly active	Roller massager	Plantar flexors	6 × 45 s 30 bpm	Much pressure as they could	1 test session	5 × 20 1-leg calf raise at BW	Yes	Foam rolling	Plantar PPT Dorsiflexion ROM Plantar MVIC Muscle morphology Muscle oxygenation pre- and post- T0, T+1 h, 24, 48, 72 h	↑ muscle tenderness at T+24 h, 48 h, 72 h NS change ROM NS change muscular performance NS change morphology NS change muscle oxygenation
Romero-Moraleda et al. (2017)	RCT	32 healthy and moderately active	High density FR	Quadriceps	5 × 60 s	Much pressure as they could	NR	5 × 20 0,5 m drop jumps	No	Neurodynamic mobilization Foam rolling	Numerical pain rating scale-10 Knee extension MVIC	Both groups : ↓ muscle pain Foam roller group ↑ muscle strength
Drinkwater et al. (2019)	RC	11 healthy young males	High density FR	Main lower limb muscles	1 × 180 s/muscle 60 bpm	Much pressure as they could	1 test session	6 × 25 eccentric knee extensors at 120°/s	Yes	Foam rolling post-T0 and before each testing point at T+24, 48, 72 h	PPT rectus femoris Mid-thigh circumference Knee flexion ROM CMJ MVIC right knee extensor	↑ muscle tenderness at T+48h NS change circumference NS change knee ROM ↑ vertical jump at 72 h NS change strength
Naderi et al. (2019)	RCT	80 healthy physically active male	High density FR	Quadriceps	4 × 120 s 30 bpm	Much pressure as they could	1 test session	4 × 25 eccentric knee extensors at 60°/s	Yes	Foam rolling post- T0, T+1, 24, 48, 72 h	Pain VAS-10 PPT Quadriceps muscle strength Joint position sense Isometric force sense pre- and post- T+1, T+24, 48, 72 h	↓ muscle pain ↑ muscle tenderness ↑ proprioception ↓ force decrements up to 48h
Romero-Moraleda et al. (2019)	RCT	38 healthy and moderately active	Vibrating FR Non-vibrating FR	Quadriceps	5 × 60 s	Much pressure as they could	NR	10 × 10 inertial flywheel eccentric squat	No	Foam rolling with vibrating roller (18Hz) Foam rolling with classic roller	Pain VAS-10 Quadriceps PPT Muscle oxygen saturation CMJ Active and passive hip extension ROM Knee flexion ROM	Vibrating > non-vibrating FR ↑ muscle tenderness ↓ pain perception ↑ passive hip extension Both FR ↑ muscle oxygenation (SmO2) ↑ vertical power ↑ active hip and knee ROM

*RC, randomized crossover; RCT, randomized controlled trial; FR, foam roller, BW, body weight; VAS, visual analogue scale; PPT, pressure pain threshold; NR, not reported; bpm, beats per minute; NS, not significant; ↑ indicates increase; ↓ indicates decrease.*

**TABLE 6:** The effects of foam rolling on range of motion.

Author (year)	Study design	Sample	Foam rolling intervention					Control	Others experimental groups	Outcome measures	Effects
			Tool(s)	Targeted area	Treatment time and rate	Intensity (VAS)	Expertise				
Peacock et al. (2015)	RC	16 athletically trained	High density FR	Main muscle of the body	1 × 30 s/muscle 10 bpm	NR	NR	No	Mediolateral plan Anteroposterior plan	SBRT	Mediolateral FR plan ↑ lower back and hamstring flexibility
Škarabot et al. (2015)	RC	11 adolescents trained swimmers	High density FR	Plantar flexors	3 × 30 s	7/10 VAS	6-months	No	Static Stretching Foam rolling Foam rolling + Static stretching	WBLT	Ankle dorsiflexion ROM FR group : NS change SS group : ↑ Combination group : ↑
Fairall et al. (2017)	RCT	12 adult amateur softball players with shoulder ROM	Lacrosse ball	Infraspinatus	2 × 60 s	NR	No	No	Foam rolling alone Static stretching alone Foam rolling + Static stretching	Glenohumeral internal rotation	FR + SS and SS > FR ↑ shoulder ROM
Le Gal et al. (2017)	RCT	11 adolescent advanced tennis players	Tennis ball	Infraspinatus and pectoralis	3 × 60 s/muscle	Much pressure as they could	NR	Yes	Foam rolling 3 times/week over 5-weeks	Glenohumeral internal rotation	↑ shoulder ROM at 5-weeks
Sağiroğlu (2017)	RC	22 well-trained soccer players	Vibrating FR Non vibrating FR	Main lower limb muscles	2 × 30 s/muscle 10 bpm	NR	No	No	Foam rolling with vibrating roller (38 Hz) Foam rolling with classic roller	SRBT	Both experimental groups : ↑ lower back and hamstring flexibility without no difference between
Guillot et al. (2019)	RCT	30 professional rugby players	High density FR	Main lower limb muscles	1 × 20–40 s/muscle 21 bpm	Much pressure as they could	1 test session	Yes	Foam rolling 1 set of 20 s 1 sets of 40 s 3 times/week over 5-weeks	Side split test Active SLR Modified Thomas test WBLT	Both FR groups : without S difference between FR groups ↑ hip ROM NS change knee ROM NS change dorsiflexion Perceived discomfort 40 s > 20 s
Oranchuk et al. (2019)	RC	22 female NCAA Division II lacrosse and soccer athletes	High density foam roller	Hamstrings	3 × 60 s 30 bpm	Much pressure as they could	1 test session	Yes	Foam rolling (FR) Superficial heating (SH) Superficial heating + Foam rolling	Passive SLR Likert scale (perceptions of efficacy)	SH, SH + FR > Control SH + FR > FR or SH ↑ hip flexion ROM SH + FR > FR but not SH more effective perception
Sullivan et al. (2013)	RCT	17 healthy and recreationnaly active	Roller-massager	Hamstrings	1–2 × 5–10 s 120 bpm	13 kg	No	Yes	Foam rolling 1 set of 5 s 2 sets of 5 s 1 set of 10 s 2 sets of 10 s	SRBT	↑ lower back and hamstring flexibility FR 10 s > FR 5 s
Halperin et al. (2014)	RC	14 healthy and recreationnaly active	Roller-massager	Plantarflexors	3 × 30 s 30 bpm	7/10 VAS	No	No	Foam rolling Static stretching	WBLT	Both groups ↑ ankle dorsiflexion ROM up to 10 min
Mohr et al. (2014)	RCT	40 subjects with less than 90° of passive hip-flexion	Bio-foam roller	Hamstrings	3 × 60 s 30 bpm	Much pressure as they could	1 test session	Yes	Foam rolling Static Stretching Combined techniques 3 times/week over 2-weeks	Passive SLR	↑ hip ROM Mixed group > FR and SS group > control
Cho et al. (2015)	RCT	50 subjects with hamstrings flexibility deficit	Wooden triangle-shaped pillow	Suboccipital	1 × 280 s	NR	NR	No	Suboccipital muscle inhibition	Finger-floor distance SLR Popliteal angle	↑ hamstrings flexibility
Grieve et al. (2015)	RCT	24 healthy subjects	Tennis ball	Sole	1 × 120 s/sole	Much pressure as they could	NR	Yes	Foam rolling	SRBT	↑ hamstring and lower back flexibility
Vigotsky et al. (2015)	RCT	23 healthy students	High density FR	Quadriceps	2 × 60 s/Slowly	NR	NR	No	Foam roller Static stretching	Modified Thomas test	↑ hip extension ROM NS change knee flexion NS change rectus femoris length
Kelly and Beardsley (2016)	RCT	26 healthy and recreationnaly active	High density FR	Plantar flexors	3 × 30 s 15 bpm	Much pressure as they could	1 test session	Yes	Foam rolling leg Control leg	WBLT pre- and post- T0, T+5, 10, 15, 20 min	↑ dorsiflexion ROM ipsilateral leg : up 20 min contraletral leg : up 10 min
Patel et al. (2016)	RCT	30 subjects with active knee extension deficit	Tennis ball	Sole	1 × 120 s/foot	Much pressure as they could	NR	Yes	Foam rolling	SRBT Active knee extension	↑ lower back and hamstring flexibility
Boguszewski et al. (2017)	RCT	37 healthy and recreationnaly active	Foam roller – not reported	Main lower limb muscles	1 × 20 min/muscle	NR	Yes	Yes	Foam rolling 2 times/week over 8-weeks	Single leg SRBT Functional Movement Screen (FMS)	↑ lower back and hamstring flexibility vs control group ↑ FMS score vs control group
Garcia-Gutiérrez et al. (2017)	RCT	33 healthy and moderately active	Vibrating FR Non-vibrating FR	Plantar flexors – dominant leg	5 × 20 s 15 bpm	Much pressure as they could	1 test session	Yes	Vibrating foam roller (32 Hz) Non-vibrating foam roller	WBLT Maximal voluntary contraction plantar flexion/dorsiflexion Both legs	Both groups ↑ ankle ROM with a crossover effect NS difference between groups NS change strength
Grabow et al. (2017)	RCT	12 healthy and recreationnaly active	Foot roller	Sole	3 × 60 s 30 bpm	7/10 VAS	NR	No	Foam rolling leg Control leg	Modified SRBT WBLT	NS change dorsiflexion ROM NS change lower and hamstrings flexibility on ipsilateral and controlateral leg
Hsuan Su et al. (2017)	RC	30 college students physically active	High density FR	Quadriceps and hamstrings	3 × 30 s/muscle	Much pressure as they could	1 test session	Yes	Static stretching Foam rolling Dynamic stretching	SRBT Modified Thomas test	↑ quadriceps and hamstring flexibility
Jung et al. (2017)	RC	22 healthy subjects	Wooden stick	Suboccipital region, hamstrings and sole	1 × 4 min/muscle	NR	No	No	Foam rolling suboccipital Foam rolling hamstrings Foam rolling sole	SRBT	Thrre groups ↑ lower and hamstrings flexibility
Do et al. (2018)	RCT	31 healthy and recreationnaly active	Micro foam roller	Plantar fascia	1 × 5 min	Much pressure as possible	NR	Yes	Foam rolling Sham group	Toe touch test Passive SLR	↑ lower back and hamstring flexibility
Killen et al. (2018)	RCT	23 healthy subjects	High density FR	Hamstrings	10 × 30 s 30 bpm	NR	1 test session	No	Static Stretching Foam rolling on dominant leg	SLR	Both groups ↑ contralateral hip ROM
Madoni et al. (2018)	RWS	22 healthy and recreationnaly active	High density FR	Hamstrings	3 × 30 s	Much pressure as they could	1 test session	Yes	Foam rolling	SLR	↑ hamstring flexibility
Phillips et al. (2018)	RC	24 healthy and recreationally active	Foam roller – not reported	Quadriceps, plantar flexors	1 × 60 s/muscle 10 bpm	Much pressure as they could	1 test session	Yes	Foam rolling : 60 s Foam rolling : 5 min Planking on a heating pad	Modified WBLT	↑ dorsiflexion ROM ↑ quadriceps flexibility 5 min > 60 s foam rolling
Jeong et al. (2019)	RC	30 young women	Massage ball	Hamstrings	3 × 30 s/zone	NR	NR	No	Foam rolling Self-stretching	90–90 SLR pre- and post- T+5, 30 min	Both groups ↑ ROM
Lim and Park (2019)	RCT	20 healthy college students	Vibrating FR Non-vibrating FR	Hamstrings	5 × 60 s	NR	NR	No	Vibrating foam roller (32 Hz) Non-vibrating foam roller Static stretching	Active SLR Active knee extension test CMJ	Vibrating > non vibrating FR ↑ hamstrings flexibility NS change vertical power
Pathania and Muragod (2019)	RCT	45 elderly subjects with hamstring flexibility deficit (65–75 years of age)	High density FRM2T blade	Hamstrings	2–3 × 100–150 s	NR	NR	No	Foam rolling (FR) Static stretching (SS) Instrument assisted soft tissue moblization (IASTM) 3 times/week over 4-weeks	Passive knee extension SRBT	↑ lower back and hamstring flexibility IASTM > FR > SS
Smith et al. (2019)	RCT	44 healthy and recreationnaly active	High density FR	Plantar flexors	3 × 30 s 60 bpm	NR	NR	No	Foam rolling Static Stretching Foma rolling + Static stretching	Ankle dorsifleixon ROM pre- and post-session 1, post- T+3, 6, 7 weeks	Three groups ↑ dorsiflexion ROM No synergic effect of FR and SS
Somers et al. (2019)	RCT	42 physical therapy students	Foam roller – not reported	Calves	2 × 60 s/Slowly	NR	NR	No	Foam rolling alone Dynamic stretch alone Foam rolling + dynamic stetch	WBLT	NS change in ankle ROM
Williams and Selkow (2019)	RC	15 healthy collegiate students	High density FR Lacrosse ball	Sole and hamstrings	1 × 120 s/muscle 60–90 bpm	As much pressure as they could	NR	No	Sole rolling Hamstrings foam Sole and hamstrings rolling	SRBT	Three techniques ↑ lower back and hamstring flexibility equally

*RC, randomized crossover; RCT, randomized controlled trial; FR, foam roller; NR, not reported; bpm, beats per minute; ↑ indicates increase; ↓ indicates decrease; SRBT, sit-and-reach box test; WBLT, weightbearing lunge test; SLR, straigth leg raise; NS, not significant.*

#### Neurophysiological Evaluations

To this end, researchers used surface electromyography (EMGs) to appreciate whether muscle activation increase was associated with better performance, and further reduced the risk of injury (Macgregor et al., 2018). EMGs were recorded non-invasively by positioning electrodes directly on a shaved skin cleaned with alcohol (Ginszt et al., 2017; Romero-Moraleda et al., 2017; Hodgson et al., 2018; Madoni et al., 2018; Beier et al., 2019; Capobianco et al., 2019; Kim et al., 2019; Mazzei, 2019; Ye et al., 2019; Table 7). Recently, the development of tensiomyography allowed rapid and reliable non-invasive investigations of the contractile properties of the skeletal muscle (Martínez-Cabrera and Núñez-Sánchez, 2016; Murray et al., 2016; Schroeder et al., 2017; Macgregor et al., 2018; Table 7).

**TABLE 7:** The effects of foam rolling on neurophysiological and physiological variables.

Author (year)	Study design	Sample	Foam rolling intervention				Control	Others experimental groups	Outcome measures	Effects
			Tool(s)	Targeted area	Treatment time and rate	Intensity				
**Neurophysiological and physiological effects**										
Martínez-Cabrera and Núñez-Sánchez (2016)	RCT	7 professional soccer players	High density FR	Rectus femoris	4 × 15 s 30 bpm	NR	No	Foam rolling leg Control leg	Muscle contractile properties (TMG)	Maintains muscle contractile properties
Murray et al. (2016)	RC	12 squash players	High density FR	Quadriceps	1 × 60 s 30 bpm	NR	Yes	Foam rolling leg Control leg	Muscle contractile properties (TMG) Superficial temperature	NS change muscle contractile properties NS change skin temperature
Casanova et al. (2017)	RC	10 athletes	Roller massager	Plantar flexors	6 × 45 s 30 bpm	NR	Yes	Foam rolling leg Control leg	Muscle oxygenation (HHb concentration) Muscle morphology	NS change muscle oxygenation NS change muscle morphology
D’Amico and Paolone (2017)	RC	16 trained males	High density FR	Main lower limb muscles per leg	1 × 30 s/muscle 6 bpm	NR	Yes	Foam rolling	Blood sample VCO2 pre- and post- each run	NS change blood lactate concentration NS change VCO2
Kalén et al. (2017)	RC	12 surf lifeguards	High density FR	Main lower limb muscles per leg	2 × 60 s	7/10	Yes	Foam rolling Running	Blood sample	Both groups ↑ blood lactate clearance
Beier et al. (2019)	RC	11 resistance trained subjects	Stick massage	Rectus femoris and gluteus maximus	1 × 120 s/muscle	Heavy pressure	No	Foam rolling Dynamic warmp-up	Muscle activation (EMGs)	NS change muscle activation
Mazzei (2019)	RCT	15 NCAA Division I swimmers	Vibrating FR Non-vibrating FR	Plantar flexors	3 × 30 s 30 bpm	NR	No	Vibrating foam rolling (1200 to 3600 rpm) Non-vibrating foam rolling	Muscle activation (EMGs)	Both groups NS change muscle activation
Kim et al. (2014)	RCT	22 healthy subjects	FR–not reported	Main lower limb muscles and back	1 × 3–6 min	NR	Yes	Foam rolling	Blood sample	Both groups : ↓ cortisol
Okamoto et al. (2014)	RC	10 healthy subjects	Polystyrene roller	Main lower limb muscles	20 repetition/muscle	NR	Yes	Foam rolling	Arterial stiffness Blood sample	↓ brachial-ankle pulse wave velocity ↑ vasoactive substance (↑ nitric oxide concentration)
Thistlethwaite et al. (2016)	Pilot test	6 subjects	PVC pipe	Iliotibial band, adductors, hamstrings, quadriceps)	1 × 180 s/muscle	NR	No	Foam rolling 3 times/week over 6 weeks	Endothelial function	↑ blood flow (↑ diameter of the femoral artery)
Ginszt et al. (2017)	Pilot test	20 healthy adults	High density FR	Right quadriceps	1 × 60 s	Much pressure as they could	No	Foam rolling leg Control leg	Muscle activation (EMGs)	↓ muscle fatigue
Hotfiel et al. (2017)	RCT	21 healthy students	High density FR	Iliotibial band	3 × 45 s	Much pressure as they could	No	Foam rolling	Arterial tissue perfusion post- T0, T+30 min	↑ arterial blood flow up to 30 min
Romero-Moraleda et al. (2017)	RCT	33 healthy and moderately active	High density FR	Quadriceps	5 × 60 s	More of their body weight	No	Foam rolling Neurodynamic mobilization	Muscle activation (EMGs) MVIC knee extension	Both interventions : ↑ muscle activation ↑ muscle strength
Schroeder et al. (2017)	RC	12 heathly and recreationnaly active	High density FR	Hamstrings, gluteus, lower back	3 × 60 s 15 bpm	40, 65, 75%	No	Weight training Stretching Foam rolling	Muscle contractile properties (TMG)	NS change muscle contractile properties
Hodgson et al. (2018)	RCT	23 healthy and recreationally active	Roller massager	Quadriceps and hamstrings	4 × 30 s/muscle 60 bpm	7/10	Yes	High frequencies (6 times/week) Low frequencies (3 times/week) over 4-weeks	Muscle activation (EMGs) Knee flexors, extensors MVIC	NS change muscle activation NS change muscle strength
Lastova et al. (2018)	RCT	15 healthy and recreationnaly active	High density FR	Main lower limb muscles and lower/upper back	1 × 40 s 15 bpm	NR	Yes	Foam rolling	Blood pressure Heart rate variability pre and post- T0, T+10, T+30 min	↓ blood pressure at 10 and 30 min ↓ sympathovagal balance at 30 min
Macgregor et al. (2018)	RC	16 healthy recreationally active males	High density FR	Quadriceps	1 × 120 s 60 bpm	6/10	Yes	Foam rolling over 3 consecutive days	Muscle activation (EMGs) Muscle contractile properties (TMG)	↓ muscle activity ↑ muscle displacement
Madoni et al. (2018)	RCT	22 healthy and recreationnaly active	High density FR	Hamstrings	3 × 30 s	Much pressure as they could	Yes	Foam rolling	Muscle activation (EMGs) Maximal knee extension/flexion	NS change muscle activation NS change strength ratio
Capobianco et al. (2019)	RC	30 young and middle-aged adults	Therapy ball	Calf	3 × 60 s/leg 15 bpm	>5/10 (discomfort level)	No	Static stretching Foam rolling + static stretching	Muscle activation (EMGs) Subcutaneous tissue thickness	↑ muscle activation (foam rolling > static stretching) NS change subcutaneous tissue thickness
Kim et al. (2019)	RC	30 participants with neck pain (age: 65.9 ± 3.4 years)	Soft inflatable rubber ball Hard massage ball	Suboccipital region	1 × 10 secs	NR	Yes	Soft inflatable rubber ball Hard massage ball	Muscle activation (EMGs) Radiography (compressed soft tissue thickness and neck extension ROM)	Soft inflatable rubber ball vs hard massage ball Less muscle activity (less muscle tension) Less compressed soft tissue thickness
Ye et al. (2019)	RC	34 healthy and physically active	High density FR	Hamstrings	10 × 30 s 30 bpm	NR	Yes	Yes	Muscle activation (EMGs) Knee flexors MVIC	NS change muscle activity and strength

*RC, randomized crossover; RCT, randomized controlled trial; FR, foam roller; NR, not reported; bpm, beats per minute; ↑ indicates increase; ↓ indicates decrease; MVIC, maximal voluntary isometric contraction; EMGs, surface electromyography; TMG, tensiomyography; NS, not significant.*

To complement the measurements made on the peripheral nervous system, other research was undertaken to increase the current understanding of the effects of FR to a more fundamental level. Researchers hypothesized that this technique was not limited to local changes in muscle tissues, but might also affect the vascular function. Some researchers collected blood analyses to measure concentrations of blood cortisol, lactate, oxytocin or even nitric oxide (Kim et al., 2014; Okamoto et al., 2014; D’Amico and Paolone, 2017; Kalén et al., 2017; Table 7). To overcome invasive constraints, they used an automatic blood pressure monitor, simple and quick to manipulate. This medical device consists of an inflatable cuff positioned on the arm. This device measures blood pressure by the oscillatory method, i.e., the heartbeats, pulse wave velocity, systolic and diastolic pressures (Okamoto et al., 2014; Lastova et al., 2018; Table 7). To achieve a more detailed analysis of both superficial and deep blood flow, ultrasound Doppler recordings were further used. This exploration is certainly more precise but requires specific knowledge in medical semiology as well as good adjustments of the device (Thistlethwaite et al., 2016; Hotfiel et al., 2017; Table 7). Formerly designed and reserved for the medical field, these advanced measurements are now gradually made available to applied research and extend current knowledge to FR. This recent spread comes in particular from a surge of recent interest from physiotherapists and rehabilitation professionals. Researchers therefore did not restrain their evaluation to functional tests for monitoring flexibility and muscle performance.

#### Short-Term Effects

Positive short-term FR effects, measured at a single-session level, were found to substantially reduce muscle pain (Schroeder and Best, 2015), in particular after exercise-induced muscle damage (Jay et al., 2014; MacDonald et al., 2014; Pearcey et al., 2015; Romero-Moraleda et al., 2017; Table 5). Regardless the tools’ use, a foam roller, a massage stick, golf or tennis balls, the FR intervention yielded immediate flexibility and ROM gains (Cheatham et al., 2015; Grieve et al., 2015; Brengesjö and Lohaller, 2017; Monteiro et al., 2017, 2019). Foam roller and roller massager, for instance, were shown to elicit comparable increased ROM (Monteiro et al., 2017, 2019). This effect would be reinforced by few additional degrees with vibrating foam rollers. Frequencies between 33 and 62 Hz would guarantee higher ROM and PPT elevations, compared to a non-vibrating foam roller (Cheatham et al., 2017, 2019; Han et al., 2017; Table 4). In addition, ROM benefits would depend on a dose-response effect of FR (120 vs. 60 s) (Monteiro et al., 2017, 2019). More surprisingly, FR would also demonstrate the ability to have a delocalized effect. For example, FR of sole or hamstring muscles might positively influence lower back and hamstring flexibility by improving the sit-and-reach test scores, with a wooden stick or a tennis ball (Grieve et al., 2015; Jung et al., 2017; Table 6).

The most supported hypothesis to explain these changes would be a modification of the autonomic nervous system responses (Joshi et al., 2018; Dębski et al., 2019; Wiewelhove et al., 2019). The slow deep pressure would induce a decrease in the tone of related skeletal motor units by stimulating mechanoreceptors. This chain reaction would elicit a parasympathetic-dominant neurophysiological state, thus eliciting a greater relaxation (Schleip, 2003b). Mechanical pressure applied during FR would elicit similar effect. For example, in participants with rounded shoulder, both trapezius and pectoralis major muscles activity decreased after a FR intervention. This progress was accompanied by a reduction in shoulder height (Choi, 2019). These findings represent a useful source of information to guide FR practitioners. Indeed, local FR treatment therefore does not seem to be limited to a single element, but may impact all surrounding structures, even the whole body. This particularity represents a particular interest for physiotherapists to correct certain postural imbalances and/or muscular flexibility deficit.

Commonly used to improve ROM, FR has also been tested during warm-up to improve the physical qualities of athletes such as muscle power, strength or agility. However, unlike ROM, performance results obtained were not always clearly established (Burk et al., 2019; Hughes and Ramer, 2019; Skinner et al., 2020). Some studies recorded improvements in muscle power, strength or agility, after a single session of FR (D’Andrea, 2016; Sağiroğlu, 2017; Stroiney et al., 2020). Other came to different conclusions. For example, FR was not shown to bring any significant improvement in performance (Burk et al., 2019). Few protocols have been tested and replicated enough to lead to real consensus. Indeed, between the protocols, many parameters differed such as outcome measures, FR tool, target muscles, type of population, participants’ FR experience, and FR instructions (e.g., pressure level, duration and rate of treatment) (Burk et al., 2019; Dębski et al., 2019). It is likely that this experimental disparity justifies the heterogeneity of the results reported in the literature. Until a reliable consensus is reached, the use of FR for warm-up should certainly not be contraindicated. In fact, unlike static stretching, there was no loss of physical capacity (Halperin et al., 2014; Bradley et al., 2016; Grabow et al., 2017; Su et al., 2017).

For the same reasons of reproducibility and the lack of sufficient data, effects of post-training FR on physiological markers remain inconclusive. In laboratory conditions, excluding physical exertion, first results revealed a decrease in brachial-ankle pulse wave velocity (from 1202 ± 105 to 1074 ± 110 cm/s) and an increase in plasma nitric oxide concentrations (from 20.4 ± 6.9 to 34.4 ± 17.2 μmol/L), reflecting an improvement in endothelial functions and arterial stiffness (Okamoto et al., 2014). Another study also observed an increase in the arterial blood flow. Compared to a baseline recording, the mean peak flow increased of 73.6% immediately after FR and 52.7% 30 min post-treatment (Hotfiel et al., 2017). The drop in blood pressure was also observed up to 30 min after FR, confirming the hypothesis of a positive effect of FR on cardiovascular protective effect and health (Lastova et al., 2018; Table 7). In training conditions, FR has been shown to be ineffective on the muscular re-oxygenation in the treated leg after a bilateral exercise inducing muscular damage (Casanova et al., 2017). Some indirect markers such as cortisol and lactate confirmed, by their fall, this positive influence of FR on blood circulation (Kim et al., 2014; Kalén et al., 2017). In conclusion, comparison of results between studies is delicate due to the singular nature of each experiment, increasing the difficulty of interpreting certain acute effects which are still undecided. Indeed, few protocols have been reproduced and sufficiently tested to establish a consensus and therefore an optimal FR program. In response to this uncertainty, two recent systematic reviews of literature proposed a framework to guide practitioners and researchers for future experiments (Dębski et al., 2019; Hendricks et al., 2019). According to these authors, acute effects identifiable by gains in ROM, increase in the PPT, reduction in DOMS, or even better vascular function, would be made possible by respecting certain rules of practice. It would therefore be wise for FR tool users to repeat each exercise 1–3 times at a rate of 20 bpm. A set of rolling should last between 30 and 120 s and followed by a 30 s recovery period. Pressure, on the other hand, has an individual character and a part of subjectivity. However, the application force should not exceed the pain tolerance threshold. On a pain scale, a 7/10 indicator or the following instruction, “*as large as possible*” is commonly used to guide practitioners for pressure (Dębski et al., 2019; Hendricks et al., 2019). FR tools, such as foam roller, roller massager or ball, should therefore be firm enough (Cheatham et al., 2015), and ischemic pressure is advised on sensitive areas until a feeling of release is obtained (Dębski et al., 2019; Hendricks et al., 2019). To complete this information, we argue that two additional parameters should be taken. To begin with, user experience in the practice of FR is required as FR requires some experience to master the level of pressure on the tool. Poor management can elicit excessive discomfort or pain, and bias the effects of FR technique. The second determining criterion is the number of areas treated. It seems that the effects of FR are short-lived, 10–20 min depending on the studies (MacDonald et al., 2013; Halperin et al., 2014; Jay et al., 2014; Škarabot et al., 2015; Kelly and Beardsley, 2016). It is therefore essential to ensure the effective duration of the routine so as not to exceed this period and risk seeing the effects dissipate in the first target areas.

#### Long-Term Interventions

The effects of FR were investigated within a span of several days or weeks. The paradigms either involved the follow-up of the effects of FR after a single session, or administered regular FR sessions along the intervention period. Protocols usually involved 2–5 sessions per week delivered within a span of 3–8 weeks. The use of a ball or foam roller demonstrated its usefulness for improving shoulder, knee, or ankle ROM (Le Gal et al., 2018; Smith et al., 2019), without harmful consequences on physical performance (Hodgson et al., 2019). Regular combination of FR and static stretching may have an additional effect for increasing ROM (Mohr et al., 2014; Škarabot et al., 2015). The effects of FR may be due to neural changes, the pressure exerting on the soft tissues increasing the tolerance to stretching, and therefore promoting performance gains (Škarabot et al., 2015).

With regard to vascular function, delayed beneficial effects were observed for a frequency of three FR sessions per week during a 6-week period (Thistlethwaite et al., 2016). A drop in blood pressure, heart rate variability, and sympathovagal balance, has also been reported (Chan et al., 2015; Lastova et al., 2018). Although this is premature to draw any firm conclusion, FR therefore appears effective to stimulate the organism and maintain better health.

### Procedures

FR techniques involve two main maneuvers which can be combined in a single routine. The pressure depends on the tool used, its density, and the target area (Miller and Rockey, 2006; Sullivan et al., 2013; Cheatham et al., 2015). Few studies recorded the pressure applied during FR with a specific designed constant pressure roller apparatus. Two experiments measured muscle strength and flexibility after a continuous pressure of 13 kg on the hamstrings (Sullivan et al., 2013), as well as 20 kg on the quadriceps, with roller massager (Bradbury-Squires et al., 2014). However, with a foam roller, these values usually range from 27 to 68% of the body weight, depending on the muscle and the position (e.g., bilateral, unilateral) of the participants (MacDonald et al., 2013; Murray et al., 2016; Macgregor et al., 2018; Baumgart et al., 2019). Research remains sparse on pressure level, and no specific value is recommended. However, the literature demonstrated the existence of a dose-dependent response to FR. Some authors reported a significant improvement between two treatments of different durations (Bradbury-Squires et al., 2014; Monteiro et al., 2017, 2017a; Phillips et al., 2018), whereas others did not corroborate an effect of the routine duration (Sullivan et al., 2013; Guillot et al., 2019; Table 6). Despite divergent results on its optimal use, FR practice systematically resulted in improved ROM and PPT (Jay et al., 2014; Cavanaugh, 2016; Kelly and Beardsley, 2016; Casanova et al., 2017; Cheatham and Baker, 2017; Garcia-Gutiérrez et al., 2018; Killen et al., 2018), trending approach consisting in the combination of active joint mobilization (e.g., knee flexion-extension movement) with FR practice (Cheatham and Baker, 2017; Cheatham et al., 2017, 2019; Cheatham and Kolber, 2018; Cheatham and Stull, 2018a, b; Table 4). Such combination provided promising early effects on ROM gains (Cheatham et al., 2017; Han et al., 2017).

For safe and effective FR interventions, literature-driven guidelines should now be outlined and conceptualized. Quantifying the biomechanical workload applied through the FR routine is of critical importance. While higher mechanical compressions on the underlying tissues might be exerted using FR, compared to MMs, a potentially harmful impact on connective tissues, nerves, vessels and bones, should not be excluded. Deleterious effects must be controlled in future designs (Fleckenstein et al., 2017). External biomechanical loads should also be quantified to determine to which extent FR differs from MM in terms of pressures. Regular practice might be more suitable than longer session durations. Prolonged FR beyond 90 s of treatment in the same area might not bring any additional benefit (Monteiro et al., 2017a), and excess may even cause harmful effects. Eventually, practitioners should certainly adjust FR practice to their own sensations. For instance, PPT should never be exceeded to prevent injuries.

## Manual Massage and Foam Rolling at the Scope of Motor Control Frameworks

### From Resemblance to Dissimilarity

MM and FR are both likely to positively affect psychometric, behavioral and physiological variables. As mentioned in the previous sections, experimental data extensively confirmed their respective beneficial effects. The same organs and tissues are targeted by both techniques, which might account for the congruent pattern of beneficial results on well-being and motor performance. Furthermore, both techniques appeared to promote motor recovery, with similar body effects during warm-up and post-exercise recovery. They are associated with increased well-being and give the opportunity to temporarily improve ROM and PPT, without altering physical performance (i.e., power, strength, agility). In practice, however, MM and FR are highly distinct. During MM, athletes have no control over the massage parameters, whereas during FR, they produce voluntary movements to complete the routine, by regulating the level of pressure exerted and the speed of execution. While this may be considered an advantage inherent to the technique, the lack of standardization of FR protocols represents an obstacle to the development of clinical applications. Indeed, the few existing guidelines do not provide medical professionals the necessary levels of reproducibility and reliability for application of such routines in clinical populations (Cheatham et al., 2015; Schroeder and Best, 2015). Classically, a MM lasts between 15 and 30 min (Standley et al., 2010), compared to 30–120 s per muscle group for FR. This difference is explained by the more holistic approach of the body during MM. More muscle groups are manually massaged, and several techniques can be used in a single area, hence increasing the whole duration of treatment. Conversely, in most FR studies, one or two muscle groups are mainly involved for 30–120 s each (Dębski et al., 2019).

### Motor Control Implications

There is currently no hypothesis or conceptual approach that distinguishes MM from FR, even though a fundamental distinction between the two types of massaging interventions stems from the nature of their requirements in terms of motor control strategies. The person receiving a MM remains passive, lying on the massage table, usually in dorsal or ventral decubitus. The routine and the pressures derive from the physiotherapists’ experience and available sensory feedback from the MM routine or provided by the patient. The gestures of the physiotherapist cannot be anticipated by the person, thus resulting in a retroactive mode of action control with an ongoing central integration of proprioceptive feedback (Hasegawa et al., 2001). Although the participant can adjust muscle tone at his convenience, he is primarily confronted with a retroactive mode of action control (Braver, 2012). Conversely, while performing FR, the person directly exerts pressures through voluntary actions to manipulate the massaging tool. FR thus requires a predominant proactive mode of action control, where proactive operations of forward and inverse modeling, which do not require sensory feedback from the periphery, are involved (Cervin et al., 2002). Although with tools such as balls or foam roller, where the individual simply uses the body weight to achieve the desired effects, it remains a comparable proactive mode of action control. If necessary, the person always has the possibility to use retroactive operations to adjust FR parameters. Contrary to MM, the sensory consequence of voluntary motor commands during FR can therefore be anticipated by means of the efferent copy derived from the forward model that associates motor commands with their sensory consequences. According to the dual mechanisms of motor control framework (Braver, 2012), MM and FR can thus be distinguished by the implementation of two distinct motor control strategies. These two interventions, with opposite modes of action, do not seem to offer any compromise. The user is unable to migrate from one mode of action to another. Interestingly, this limit could be easily resolved with technological advances and the appearance of collaborative robots. By its unique functionalities, this new generation of robot might conceptually reconcile the opposition of MM and FR. Indeed, the autonomy and real-time interaction capabilities of these robots with the user offer new perspectives in terms of motor control that have yet to be explored.

## Robotic Massage: An Emerging Paradigm

### The Instrumentalization of Massage

To the best of our knowledge, there is yet no massage technique allowing for an actual combination of the retroactive and proactive modes of action control. The advent of intelligent robotic massage might contribute to integrate these two types of motor control within single massaging routines, and therefore provide a unique and complete approach to the massage experience. Spurred by an economic purpose, and to overcome limits of human faculties, several assisting devices offering massage programs are already available in beauty salons and physiotherapists’ offices (e.g., LPG endermologie^®^, H2O Body system^®^, wellsystem^TM^). Such devices offer a relevant alternative to the MM, due to the fatigue of the physiotherapist, and the loss of efficiency resulting from repetitive practice. Accordingly, many professionals suffer from pathologies such as musculoskeletal disorder due to the amount of exposure to repeated uncomfortable postures, maneuvers and manipulations (Albert et al., 2008; Murali et al., 2014; Rossettini et al., 2016). Another strength of robotic devices delivering massages is the opportunity to homogenize massage techniques, such as the palpate-rolling in the context of anticellulite (Xiaoqin and Yonggen, 2010; Mezencevová et al., 2017). In the case of a purely robotic massage routine, participants for now remain in a passive situation, and thus exclusively engage retroactive modes of action control. Despite obvious advantages, first robotic devices failed to provide interactivity options of users toward the system, still requiring the presence and action of a physiotherapist, and remain somewhat expensive.

### The Limits of Standardization

Following pain and pathologies linked to the practice of MM for physiotherapist, a second wave of full automated systems has rapidly emerged. Although apparently similar, these tools were distinct by the substitution of the physiotherapist by a qualified operator specific to each machine. Unlike other devices, once turned on, some of these apparatus operate independently and therefore no longer required the intervention of a third person. Unfortunately, a standardized protocol was implemented without providing real adjustments to/by the user. Currently, several commercials devices, such as intermittent sequential pneumatic (Zelikovski et al., 1993), warm underwater water-jet massages (Viitasalo et al., 1995), and whole body vibrations (Edge et al., 2009), are thus available (Poppendieck et al., 2016). Unlike MM, during which the physiotherapist may encounter difficulties in applying constant pressures, techniques and durations, these devices providing whole body vibrations allow a deep control of vibration frequency, duration, and amplitude (Edge et al., 2009). In practice, these devices demonstrated limited effectiveness on physical performance (e.g., running performance, strength) and inconclusive results on recovery and physiological markers such as creatine kinase activity, pH, and lactate after intense effort (Zelikovski et al., 1993; Edge et al., 2009; Lau and Nosaka, 2011). We assume that individualization of the massage, incorporating morphology and expectations of the user, would certainly provide greater benefits. While some devices allow users to adjust few parameters of the massage, such modifications are not yet ergonomic (Golovin et al., 2018). Likewise, adjusting the program remains often impossible once the routine initiated. In all cases, the interaction between the user and the device therefore remains restricted to a retroactive motor control strategy for the user.

### The Challenges of Robotics

Advances in robotics gradually spread within the field of medicine (Petrescu et al., 2016). Like the da Vinci surgical robot and the ROSA^TM^ spine robot, it is clearly established that robots can assist surgeons with precision during minimally invasive procedures such as arthroscopy or laparoscopic splenectomy (Chapman et al., 2002; Lopez et al., 2013; Lefranc and Peltier, 2016). The increasing amount of robotic intervention should be considered within a broader framework. Indeed, once purely human, several medical interventions now seem to involve increasing amounts of robotic solutions. Massage interventions, for instance, could be envisioned as part of a continuum extending from purely robotic to purely human interventions. Nonetheless, robotic solutions remain insufficient and suffer from several limitations, particularly in terms of individualization, that hampers their therapeutic relevance in the absence of medical supervision. Due to the complexities and specificities of clinical interventions, it seems difficult to purely replace the massage delivered by a physiotherapist by a robot. There are multiple degree of adjustments to the patient’s characteristics that remain difficult (not to say impossible) to implement in a robot. For these reasons, innovation in terms of robotic massages quickly spread to the field of wellness, less demanding and easier to reproduce. In this context, massage robots can be regarded as an important support, albeit emphasizing that the robot remains a tool, and not a possible avatar that could substitute actual human interventions.

Two similar projects, one Russian in 1998^1^, and a second in Israel, developed back massage robots, but failed to move from the project stage to commercial production (Nissim, 2001). In Japan, the Waseda University and Asahi Roentgen company developed the Waseda Asahi Oral-Rehabiliation Robot 1 (WAO-1) (Takanishi et al., 2008, 2009). This robotic device, originally designed to promote recovery of temporomandibular disorders, was highly technical, very expensive, and targeted a specific population. Experiments provided useful information on the pressure exerted on masseter and temporal muscles, with values ranging from 100 g to 1.5 kg (Koga et al., 2008; Ariji et al., 2009a, b, 2010; Ishii et al., 2009; Obokawa et al., 2009; Solis et al., 2009; Hiraiwa et al., 2013; Table 8). The most effective pressure inducing a better easy-mouth opening was 800 g on small facial muscles. This robot also increased perceived comfort of users, muscle pain management, increased perceived heat, and promoted functional motor recovery (e.g., mouth opening, blood circulation, saliva production, muscle thickness (Koga et al., 2008; Ariji et al., 2009a, b, 2010, 2015, 2016; Ishii et al., 2009; Obokawa et al., 2009; Solis et al., 2009; Hiraiwa et al., 2013; Table 8). Other similar robotics projects provided promising results with a drop in lumbar strain, heart rate and muscle activity (Peng et al., 2010; Luo and Chang, 2011; Hu et al., 2013; Table 8). Finally, a robotic massage system achieved attractive performance on relaxation by a respective decrease and increase of beta and alpha powers (Luo et al., 2016; Table 8). However, due to the small sample sizes and the complex study design, more experimental investigation remained needed to validate these results. A critical strength of these automated devices is the autonomy and the lack of external intervention of a physiotherapist, but they do still not allow individualizing the massage *per se*. For instance, the intensity of the massage did not integrate the pain threshold tolerated by the user and the preprogrammed trajectories did not really adapt to the morphology of each person. The available robotic massages therefore positioned the user in a passive situation, once again favoring a retroactive control mode, similar to that elicited by the MM. Furthermore, by treating a single relatively restricted area, the robot ended up repeating the same trajectories. In view of these circumstances, the lack of human/robot interaction prevents from real anticipation of the massage maneuvers by the user, hence reducing a switch from a retroactive to a proactive control mode. For these reasons, and given the high cost of these solutions, such devices have not yet reached the market.

**TABLE 8:** Different acute effects of robotic massage.

Author (year)	Study design	Sample	Robotic massage intervention					Control	Others experimental groups	Test – outcome measures	Effects
			Robot	Targeted area	Techniques	Treatment time	Intensity				
Koga et al. (2008)	CCT	Head model 11 healthy subjects	WAO-1	Masseter and temporalis	Effleurage, petrissage (rotation and rubbing)	1 × 1–5 min/parotid gland	1–10 N	No	Robotic massage Manual massage	Level of force Saxon test Skin temperature Size of masseter muscle	Both groups Same force production ↑ production of saliva(doctor > robot) ↑ skin temperature ↑ size of muscle
Obokawa et al. (2009)	CCT	29 healthy subjects 26 subjects with TMJ disorders	WAO-1	Masseter and temporalis	Effleurage, petrissage (rotation and rubbing)	1 × 10 min	1–10 N	No	Robotic massage 1 ∼ 2 N 6 ∼ 8 N 10 N 6–10 N	Comfort VAS-10 Perceived warmth VAS-10 Easy-mouth opening VAS-10 Perceived lameness VAS-10	Four groups ↑ comfort ↑ perceived warmth 6–10 N : ↑ easy-mouth opening ↑ lameness muscle
Ishii et al. (2009)	CCT	18 healthy subjects	WAO-1	Masseter and temporalis	Effleurage, petrissage (rotation and rubbing)	1 × 120 s/parotide gland	5–15 N	No	Robotic massage Manual massage	Saxon test Skin temperature Width of masseter muscle	Both groups ↑ production of saliva ↑ fascial skin temperature ↑ width muscle
Ariji et al. (2009a)	CCT	6 healthy subjects 6 subjects with TMJ disorders	WAO-1	Masseter and temporalis	Effleurage, petrissage (rotation and rubbing)	7 × 60 s/muscle	1–12 N	No	Robotic massage 1 ∼ 2 N 6 ∼ 8 N 10 N 3 times/2 week over 6–22 weeks	Pain VAS-10 Impediments of daily life VAS-10 Perceived comfort VAS-10 Perceived warmth VAS-10 Easy-mouth opening VAS-10	↓ muscle pain ↑ daily life 6 ∼ 8 N: ↑ comfort 6–10 N: ↑ warmth 10 N: ↑ easy-mouth opening
Ariji et al. (2009b)	CCT	16 healthy subjects 2 subjects with TMJ disorders	WAO-1	Masseter and temporalis	Effleurage, petrissage (rotation and rubbing)	7 × 60 s/muscle	1–12 N	No	1 ∼ 2 N 6 ∼ 8 N 8–10 N 10 N 3 times/2 weeks	Pain VAS-10 Easy-mouth opening VAS-10 Perceived comfort VAS-10 Masseter stiffness index	↓ muscle pain ↑ easy mouth opening 6–10 N: ↑ comfort 6–10 N: ↑ muscle stiffness index
Solis et al. (2009)	CCT	12 healthy subjects	WAO-1 WAO-1R	Masseter and temporalis	Effleurage, petrissage (rotation and rubbing)	1 × 60 s/muscle	3–12 N	No	Robotic massage WAO-1 WAO-1R	Time index Masseter thickness Mouth opening index Skin temperature	WAO-1R > WAO ↓ require time ↑ muscle thickness Trend ↑ mouth opening NS change skin temperature
Ariji et al. (2010)	CCT	15 subjects with single or bilateral TMJ disorders	WAO-1	Masseter and temporal	Effleurage, petrissage (rotation and rubbing)	7–10 × 60 s/muscle	8–12 N		Unilateral robotic massage Bilateral robotic massage 3 times/week over 6-weeks	Masseter thickness Intramuscular sonographic appearence Pain VAS-10 Perceived comfort VAS-10 Perceived warmth VAS-10 Easy-mouth opening VAS-10	↓ muscle thickness for symptomatic side ↓ muscle pain ↓ anaeochoic areas ↑ comfort ↑ warmth ↑ easy-mouth opening
Peng et al. (2010)	RCT	1 healthy man	Ronot – not reported	Back muscles	Pushing, picking-up and kneading	NR	NR	No	Robotic massage	Heart rate Breathing rate Skin temperature	↓ heart rate NS change breathing rate NS change skin temperature
Luo and Chang (2011)	RCT	5 healthy adults	Multi-finger robot hand	Shoulder	Grasp-kneading	1 × 10 min	1–20 N	Yes	Hand massage Robot hand massage	Muscle activity	Both groups ↓ muscle activity
Hiraiwa et al. (2013)	RCT	16 patients with myofascial pain 24 healthy subjects	WAO-1	Masseter and temporal	Effleurage, petrissage (rubbing movement)	7–10 × 60 s/muscle	6–14 N	Yes	Patients1 time/week or 2 weeks over 6–12 weeks	Masseter PPT Pain VAS-10 Impediments of daily life VAS-10 Perceived comfort VAS-10 Perceived warmth VAS-10 Easy-mouth opening VAS-10 every 3 sessions	↓ sensivity only in patients ↓ muscle pain ↑ comfort ↑ warmth ↑ easy-mouth opening
Lei Hu et al. (2013)	RCT	30 patients with lumbar muscle strain	NR	Latissimus dorsi and erector spinae	Rolling, thumb kneading, pinching, pressing and vibrating	3* techniques with interval about 5 min	NR	No	Robotic massage (based on the expert database)	Lumbar PPT Lumbar strain VAS-10	↓ lumbar sensitivity ↓ lumbar strain
Ariji et al. (2015)	RCT	41 patients with temporomandibular disorder	WAO-1	Masseter and temporalis	Effleurage, petrissage (rotation and rubbing)	7 × 60 s/muscle	6–14 N	No	Effective group Ineffective group 5 times/2 weeks over 12 weeks	Muscle thickness Pain VAS-10 Maximal mouth opening Impediments of daily life VAS-10 Perceived comfort VAS-10 Perceived warmth VAS-10 Easy-mouth opening VAS-10 all 3 sessions	↓ muscle thickness symptomatic ↓ muscle pain ↑ maximal mouth opening ↑ daily life ↑ comfort ↑ warmth ↑ easy-mouth opening
Ariji et al. (2016)	RCT	37 patients with TMJ disorders	WAO-1	Masseter and temporalis	Effleurage, petrissage (rotation and rubbing)	7 × 60 s/muscle	10 N	No	Effective group Ineffective group 5 times/2 weeks over a median duration of 9.5 weeks	Muscle thickness Pain VAS-10 Maximal mouth opening pre- and post-treatment T0, T+1, 5 weeks	↑ frequency of visibility of the distinct intramuscular echo genic bands ↓ elasticity index ratio ↑ maximal mouth opening
Luo et al. (2016)	RCT	7 healthy sibjects	Robot – not reported	Back	Pressing, rubbing and stroking	1 × 10 min	NR	No	Massage robotic	Electrical activity of the brain pre- and post- treatment	↑ delta power ↓ alpha power (suggesting relaxation)

*RCT, randomized controlled trial; CCT, controlled clinical trial; TMJ, temporomandibular joint; WAO1-R, Waseda Asahi Oral Rehabilitation Robot 1; N, Newton; NR, not reported; ↑indicates increase; ↓, indicates decrease; NS, not significant.*

### The Perspectives of an Interactive and Autonomous Robotics

In this review, we shall consider a conceptual approach intended as a preamble to an original and timely research topic. Beyond a conceptual comparison, we question the possibility of an emerging solution to really match the two modes of control of the participant receiving the massage. Through incorporation of sensors into each axis, the last generation of devices, called collaborative robots, offer promising and innovative solutions which might definitively resolve the main issues mentioned previously. A Singaporean startup (AiTreat) developed since 2015 a device, called EMMA, specialized in the ‘Tui Na’ therapeutic massage. Although this massage robot uses some principles of artificial intelligence to customize massage trajectories, it is not entirely autonomous. The machine still requires the intervention of a health professional to identify the treatment areas and set the robot (Qiu et al., 2019). A Spanish company, Adamo Robot, then developed a physiotherapeutic robotic treatment solution since 2015. This device has the particularity of operating with compressed air, therefore without direct contact with the user (Jimenez, 2019). In the same massage robotic field, Massage Robotics, an American startup, developed a concept dedicated to massage centers, but the patent accompanying this project has been abandoned (Mackin, 2017). Indeed, based on a patent already existing in the field, their patent was refused after an analysis by a validation office, due to a lack of innovative character (Nissim, 2001). Finally, Capsix Robotics, a French startup, developed since 2016 a solution with a iYU^®^ robot, intended for full autonomy and back muscle relaxation. The robotic device, equipped with a 3D sensor and coupled to its generic model, is expected to reproduce trajectories on any type of morphology (Eyssautier and Gibert, 2018). These different projects represent emerging solutions to make massages accessible to as many people as possible in the gym or workplace, for example. However, to our knowledge, no study has been carried out to demonstrate the effectiveness of massages provided by such collaborative robot. This issue may be critical in ageing population. Japan is the first concerned with the highest rates of aging (Anderson and Hussey, 2000; Koga et al., 2008), and the country anticipates an increased in needs for medical care (Koga et al., 2008). Robotic solutions are scalable and robotic devices allow non-therapeutic and therapeutic interventions with standardized routines, including automatic adaptation to the morphology of the person through preliminary configuration recordings. Interestingly, the most recent robotic solutions also allow for manual control to adjust the pressure applied by the device in real-time.

To our knowledge, iYU^®^ robot is the first device allowing users to implement such options, hence conceptually conciliating the proactive and retroactive modes of action control. From a conceptual viewpoint, such advent in robotic solutions might be a pioneering birth for a new kind of massages combining these two modes of action control. A major innovation of such devices would offer the opportunity to navigate from automated massage routines to user-controlled massage routines. When the robot operates based on a predetermined massage mode selected by the user, a primary retroactive motor control strategy would be engaged as long as the trajectories cannot be predicted by the participant, i.e., such as during a MM. By interacting with the machine and controlling the parameters of the massage with a remote-control device, the user could switch to a proactive mode of action control, such as during FR. This transition faculty, from one mode of control to the other, definitely represents an innovative conceptual approach in terms of human-machine interactions.

### Limits and Perspectives

To date, with the development of collaborative robotic massage, it is possible to see a novel stochastic sensorimotor user experience. These robots are now less expensive and provide multiple advantages for users such as increased precision, availability, privacy and user choices. The addition of a remote user control, as in the case of iYu^®^ Pro, would complete the device and the whole extent of possible fields. In this context, an opportunity to interact with the device during the session would be offered, reconciling the two motor control strategies. Although these emerging solutions and the idea of conceptual bridge are attractive, extreme caution should be exercised and critical aspects questioning both users’ safety and benefits of the massage are awaiting experimental investigation. This freedom of use certainly offers new perspectives, but also implies special attention to the level of autonomy granted, so as not to represent any risk for users. Pressure levels and/or trajectories cannot be randomly administered, and these parameters should be predetermined and supervised by a professional. Also, therapeutic massages require consideration of the histological structure and physiological processes occurring in the body (Lima et al., 2020). This aspect represents a major issue since these limits are different from one person to another, depending on the muscle mass, the habit of self-massage and the presence of dysfunctions requiring medical considerations. For both safety and individualization reasons, purely replacing therapeutic massage interventions classically administered by physiotherapists by a robotic device appears impossible. It is indeed necessary to obtain feedback during the massage routine, directly from the patient and as a result of palpation performed by the therapist. Nonetheless, new types of robotic massages should continue being tested in experimental studies, in particular to differentiate the effects of robotic massages in a so-called preventive/wellness context, from those encountered in a therapeutic context.

In addition, from an ethical standpoint, robotic replicates of MM could easily be considered an inappropriate substitute to actual human interventions. Nonetheless, this should not condemn the potential relevance of robotic solutions. These offer many advantages, particularly from a preventive viewpoint. Considering the important workload for physiotherapists and frequent limited medical resources, robotic devices represent a solid alternative to provide *assistance* in their tasks (Golovin et al., 2018). They may alleviate part of the workload faced by medical professionals, and be used to administer simple and reproducible preliminary manipulations. A compound benefit would be allowing physiotherapists to focus on more complex and demanding interventions which cannot be performed by a robot (e.g., joint mobilization, stretching and strengthening exercises). For instance, a collaborative and intelligent robot could be an efficient tool to reduce the risk of musculoskeletal injuries associated with the lack of joint mobilization (e.g., thumb, wrist, shoulder, neck and low back pain). Robotic solutions would also provide an opportunity to democratize access to the practice of massage or self-massage, and thus contribute to improve well-being, health and therapeutic outcomes (Golovin et al., 2018).

Nonetheless, the use of a robotic device for curative massage interventions is questionable. It raises the issue of whether the actual experience of a physiotherapist can be robotized. Physiotherapists adjust their manipulations based on the reaction of their patient. Apart from the massage of scar tissue, MM requires intense and repetitive work by the practitioner. The fine adjustments derived from ongoing feedback, which are continuously available to the physiotherapist, remain essential to the treatment efficacy. MM remains, in this view, a specific form of human interaction that cannot be restricted to mechanical pressures, and robots remain, at this point, a tool at the disposal of medical practitioners.

Eventually, the presence of a remote control allowing to interact in real-time with the robotic device during the massage may hamper the benefits of the massage. For instance, in a clinical context, the patient could avoid the necessary amount of pain associated with pain in edema resorption routines. Also, each control command of the user on the remote requires cognitive operations. Requirements for cognitive control during the massage might preclude optimal states of relaxation. During MM, the user usually manages to detach himself from the effects of the routine by practicing the so-called “letting go” (Corner et al., 1995; Richards, 1998). Among the main unresolved questions, researchers still have to determine whether users are able to relax despite the cognitive mobilization required by the remote control and the interaction with the device. Researchers should also question whether a robotic massage may perceived as being as effective as a MM performed by a physiotherapist for wellness purposes, whether its benefits are similar to those of FR or MM, and whether it may influence psychological (e.g., perceived relaxation, fatigue, pain) and physiological variables (e.g., decrease in perceived anxiety, decreased arousal) in a similar way. Resolving these issues will undoubtedly be an exciting focus of research in the coming years.

## Author Contributions

YK, AG and FDR designed the conceptual background of the review and wrote the manuscript. YK, CE, AG, and FDR read, amended, and approved the final version.

## Conflict of Interest

CE was employed by the company Capsix Robotics. The remaining authors declare that the research was conducted in the absence of any commercial or financial relationships that could be construed as a potential conflict of interest.

## Footnotes

1 http://medicalrobot.narod.ru/articles.html

## References

[B1] Abels K. M. (2013). *The Impact of Foam Rolling on Explosive Strength and Excitability of the Motor Neuron Pool: Materials Science.* Austin, TX: The University of Texas at Austin.

[B2] Aboodarda S. Spence A. Button D. C. (2015). Pain pressure threshold of a muscle tender spot increases following local and non-local rolling massage. *BMC Musculoskelet. Disord.* 16:265. 10.1186/s12891-015-0729-5 26416265 PMC4587678

[B3] Abrantes R. Nunes S. Monteiro E. Fiuza A. Cesar Cunha J. Ribeiro M. (2019). Massage acutely increased muscle strength and power force. *J. Exerc. Physiol. Online* 22 100–109.

[B4] Albert W. J. Currie-Jackson N. Duncan C. A. (2008). A survey of musculoskeletal injuries amongst Canadian massage therapists. *J. Bodywork Movement Ther.* 12 86-93. 10.1016/j.jbmt.2007.03.003 19083660

[B5] Anderson G. F. Hussey P. S. (2000). Population aging: a comparison among industrialized countries. *Health Aff* 19 191-203. 10.1377/hlthaff.19.3.191 10812799

[B6] Andrade C. K. (2013). *Outcome-Based Massage: Putting Evidence into Practice.* Philadelphia, PA: Lippincott Williams and Wilkins.

[B7] Arabaci R. (2008). Acute effects of pre-event lower limb massage on explosive and high speed motor capacities and flexibility. *J. Sports Sci. Med.* 7 549-555. 24149965 PMC3761914

[B8] Arazi H. Asadi A. Hoseini K. (2012). Comparison of two different warm-ups (static-stretching and massage): effects on flexibility and explosive power. *Acta Kinesiol.* 6 55–59.

[B9] Ariji Y. Katsumata A. Hiraiwa Y. Izumi M. Iida Y. Goto M. (2009a). Use of sonographic elastography of the masseter muscles for optimizing massage pressure : a preliminary study. *J. Oral Rehabil.* 36 627–635. 10.1111/j.1365-2842.2009.01977.x 19602100

[B10] Ariji Y. Katsumata A. Hiraiwa Y. Izumi M. Sakuma S. Shimizu M. (2010). Masseter muscle sonographic features as indices for evaluating efficacy of massage treatment. *Oral Radiol. Endodontol.* 110 517-526. 10.1016/j.tripleo.2010.05.003 20868996

[B11] Ariji Y. Katsumata A. Ogi N. Izumi M. Sakuma S. Iida Y. (2009b). An oral rehabilitation robot for massaging the masseter and temporal muscles : a preliminary report. *Oral Radiol.* 25 53-59. 10.1007/s11282-009-0014-0

[B12] Ariji Y. Nakayama M. Nishiyama W. Ogi N. Sakuma S. Katsumata A. (2015). Potential clinical application of masseter and temporal muscle massage treatment using an oral rehabilitation robot in temporomandibular disorder patients with myofascial pain. *CRANIO^®^* 33 256-262. 10.1179/2151090314Y.0000000030 26714800

[B13] Ariji Y. Nakayama M. Nishiyama W. Ogi N. Sakuma S. Katsumata A. (2016). Can sonographic features be efficacy predictors of robotic massage treatment for masseter and temporal muscle in patients with temporomandibular disorder with myofascial pain? *CRANIO^®^* 34 13-19. 10.1179/2151090314Y.0000000037 25399824

[B14] Arroyo-Morales M. Olea N. Martínez M. M. Hidalgo-Lozano A. Ruiz-Rodríguez C. Díaz-Rodríguez L. (2008). Psychophysiological effects of massage-myofascial release after exercise : a randomized sham-control study. *J. Alternat. Compl. Med.* 14 1223-1229. 10.1089/acm.2008.0253 19123877

[B15] Baumgart C. Freiwald J. Kühnemann M. Hotfiel T. Hüttel M. Hoppe M. W. (2019). Foam rolling of the calf and anterior thigh : biomechanical Loads and acute effects on vertical jump height and muscle stiffness. *Sports* 7:27. 10.3390/sports7010027 30669477 PMC6359537

[B16] Beier Z. Earp I. Korak J. A. (2019). Self-myofascial release does not improve back squat range of motion, alter muscle activation, or aid in perceived recovery 24-hours following lower body resistance training. *Int. J. Exerc. Sci.* 12 839-846. 31156751 10.70252/GTDB7892 PMC6533090

[B17] Best T. M. Hunter R. Wilcox A. Haq F. (2008). Effectiveness of sports massage for recovery of skeletal muscle from strenuous exercise. *Clin. J. Sport. Med.* 18 446–460. 10.1097/jsm.0b013e31818837a1 18806553

[B18] Boguszewski D. Falkowska M. Adamczyk J. G. Białoszewski D. (2017). Influence of foam rolling on the functional limitations of the musculoskeletal system in healthy women. *Biomed. Hum. Kinet.* 9 75-81. 10.1515/bhk-2017-0012

[B19] Bradbury-Squires D. J. Noftall J. C. Sullivan K. M. Behm D. G. Power K. E. Button D. C. (2014). Roller-massager application to the quadriceps and knee-joint range of motion and neuromuscular efficiency during a lunge. *J. Athletic Train.* 49 133-140. 10.4085/1062-6050-49.5.03 25415414 PMC4495431

[B20] Bradley J. Gomez J. Woods T. (2016). The effect of foam rolling on subsequent exercise performance in man. *Sec. Biomed. Sci.* 185 S45-S45.

[B21] Braver T. S. (2012). The variable nature of cognitive control: a dual mechanisms framework. *Trends Cogn. Sci.* 16 106–113. 10.1016/j.tics.2011.12.010 22245618 PMC3289517

[B22] Brengesjö O. Lohaller J. (2017). Effects of foam rolling on ankle joint ROM and hamstring flexibility. *Open Orthop. J.* 9 450–455.

[B23] Brummitt J. (2008). The role of massage in sports performance and rehabilitation : current evidence and future direction. *North Am. J. Sports Phys. Ther.* 3 7-21. PMC2953308 21509135

[B24] Burk C. Perry J. Lis S. Dischiavi S. Bleakley C. (2019). Can myofascial interventions have a remote effect on ROM? a systematic review and meta-analysis. *J. Sport Rehabil.* 1 1-23. 10.1123/jsr.2019-0074 31629335

[B25] Calvert R. N. (2002). *The History of Massage : An Illustrated Survey From Around the World.* Rochester: Inner Traditions-Bear and Co.

[B26] Capobianco R. A. Mazzo M. M. Enoka R. M. (2019). Self-massage prior to stretching improves flexibility in young and middle-aged adults. *J. Sports Sci.* 37 1543-1550. 10.1080/02640414.2019.1576253 30714484

[B27] Carcano Y. Isembrand B. Wieczorek G. Boudjemaa B. (2010). Le ressenti de sportifs lors d’un massage de récupération en termes de douleur et fatigue musculaires et de bien-être. *Rôle Place Bandages Adhésifs Actifs Coule.* 10 46-50. 10.1016/S1779-0123(10)74907-X

[B28] Casanova N. Reis J. F. Vaz J. R. Machado R. Mendes B. Button D. C. (2017). Effects of roller massager on muscle recovery after exercise-induced muscle damage. *J. Sports Sci* 36 56-63. 10.1080/02640414.2017.1280609 28095747

[B29] Cavanaugh T. (2016). *The Effects of Foam Rolling on Muscular Co-activation Around the Knee Joint. Master of Science in Kinesiology.* St. John’s, NL: Memorial University of Newfoundland.

[B30] Ceca D. Elvira L. Guzmán J. F. Pablos A. (2017). Benefits of a self-myofascial release programme on health-related quality of life in people with fibromyalgia : a randomized controlled trial. *J. Sports Med. Phys. Fitness* 57 993–1002. 28139112 10.23736/S0022-4707.17.07025-6

[B31] Cervin A. Eker J. Bernhardsson B. Årzén K.-E. (2002). Feedback–feedforward Scheduling of control tasks. *Real Time Syst.* 23 25-53. 10.1023/A:1015394302429

[B32] Chan Y.-C. Wang T.-J. Chang C.-C. Chen L.-C. Chu H.-Y. Lin S.-P. (2015). Short-term effects of self-massage combined with home exercise on pain, daily activity, and autonomic function in patients with myofascial pain dysfunction syndrome. *J. Phys. Ther. Sci.* 27 217-221. 10.1589/jpts.27.217 25642077 PMC4305566

[B33] Chapman W. H. H. Albrecht R. J. Kim V. B. Young J. A. Chitwood W. R. (2002). Computer-assisted laparoscopic splenectomy with the da VinciTM surgical robot. *J. Laparoend. Adv. Surg. Tech.* 12 155-159. 10.1089/10926420260188038 12184899

[B34] Cheatham S. W. Baker R. (2017). Differences in pressure pain threshold among men and women after foam rolling. *J. Bodywork Mov. Ther.* 21 978-982. 10.1016/j.jbmt.2017.06.006 29037655

[B35] Cheatham S. W. Kolber M. J. (2018). Does roller massage with a foam roll change pressure pain threshold of the ipsilateral lower extremity antagonist and contralateral muscle groups? An exploratory study. *J. Sport Rehabil.* 27 165-169. 10.1123/jsr.2016-0196 28253066

[B36] Cheatham S. W. Kolber M. J. Cain M. (2017). Comparison of video-guided, live instructed, and self-guided foam roll interventions on knee joint range of motion and pressure pain threshold: a randomized controlled trial. *Int. J. Sports Phys. Ther.* 12 242-249. 28515979 PMC5380867

[B37] Cheatham S. W. Kolber M. J. Cain M. Lee M. (2015). The effects of self-myofascial release using a foam roll or roller massager on joint range of motion, muscle recovery, and performance: a systematic review. *Int. J. Sports Phys. Ther.* 10 827-838. 26618062 PMC4637917

[B38] Cheatham S. W. Stull K. R. (2018a). Comparison of three different density type foam rollers on knee range of motion and pressure pain threshold: a randomized controlled trial. *Int. J. Sports Phys. Ther.* 13 474-482. 30038833 PMC6044602

[B39] Cheatham S. W. Stull K. R. (2018b). Comparison of a foam rolling session with active joint motion and without joint motion: a randomized controlled trial. *J. Bodywork Movement Ther.* 22 707-712. 10.1016/j.jbmt.2018.01.011 30100300

[B40] Cheatham S. W. Stull K. R. Kolber M. J. (2019). Comparison of a vibration roller and a nonvibration roller intervention on knee range of motion and Pressure pain threshold: a randomized controlled trial. *J. Sport Rehabil.* [Epub ahead of print]. 10.1123/jsr.2017-0164 28787233

[B41] Cho S.-H. Kim S.-H. Park D.-J. (2015). The comparison of the immediate effects of application of the suboccipital muscle inhibition and self-myofascial release techniques in the suboccipital region on short hamstring. *J. Phys. Ther. Sci.* 27 195-197. 10.1589/jpts.27.195 25642072 PMC4305561

[B42] Choi J.-H. (2019). Effect of an exercise program using a foam roller on shoulder height and muscle activity in adults in their twenties with round shoulder. *Indian J. Public Health Res. Dev.* 10 1112-1118. 10.5958/0976-5506.2019.01220.8

[B43] Corner J. Cawley N. Hildebrand S. (1995). An evaluation of the use of massage and essential oils on the wellbeing of cancer patients. *Int. J. Palliat. Nurs.* 1 67-73. 10.12968/ijpn.1995.1.2.67 29323588

[B44] Correira P. P. (2016). Effects of roller massager on muscle performance, morphology, and oxygenation after exercise-induced muscle damage. *J. Sports Sci* 36 56-63. 10.1080/02640414.2017.1280609 28095747

[B45] Crane J. D. Ogborn D. I. Cupido C. Melov S. Hubbard A. Bourgeois J. M. (2012). Massage therapy attenuates inflammatory signaling after exercise-induced muscle damage. *Sci. Transl. Med.* 4:119ra13. 10.1126/scitranslmed.3002882 22301554

[B46] Cupido C. (2010). *Effects of Massage Therapy After Exhaustive Endurance Exercise in Young Healthy Males.* Masters of Science in Kinesiology Thesis. Degree of Masters of Science in Kinesiology, McMaster University, Hamilton, ON.

[B47] D’Amico A. Paolone V. (2017). The effect of foam rolling on recovery between two eight hundred metre runs. *J. Hum. Kinet.* 57 97-105. 10.1515/hukin-2017-0051 28713462 PMC5504582

[B48] D’Andrea J. (2016). *Foam Rolling as a Novel Warm-up Technique for Anaerobic Power activities.* Wayne, NJ: The William Paterson University of New Jersey.

[B49] Dawson L. G. Dawson K. A. Tiidus P. M. (2004). Evaluating the influence of massage on leg strength, swelling, and pain following a half-marathon. *J. Sports Sci. Med.* 3:37. PMC3990931 24778552

[B50] Dębski P. Białas E. Gnat R. (2019). The parameters of foam rolling, self-myofascial release treatment: a review of the literature. *Biomed. Hum. Kinet.* 11 36–46. 10.2478/bhk-2019-0005

[B51] Delextrat A. Calleja-González J. Hippocrate A. Clarke N. D. (2013). Effects of sports massage and intermittent cold-water immersion on recovery from matches by basketball players. *J. Sports Sci.* 31 11-19. 10.1080/02640414.2012.719241 22935028

[B52] Do K. Kim J. Yim J. (2018). Acute effect of self-myofascial release using a foam roller on the plantar fascia on hamstring and lumbar spine superficial back line flexibility. *Phys. Ther. Rehabil. Sci.* 7 35-40. 10.14474/ptrs.2018.7.1.35

[B53] Drinkwater E. J. Latella C. Wilsmore C. Bird S. P. Skein M. (2019). Foam rolling as a recovery tool following eccentric exercise: potential mechanisms underpinning changes in jump performance. *Front. Physiol.* 10:768. 10.3389/fphys.2019.00768 31297062 PMC6607216

[B54] Drust B. Atkinson G. Gregson W. French D. Binningsley D. (2003). The effects of massage on intra muscular temperature in the vastus lateralis in humans. *Int. J. Sports Med.* 24 395–399. 10.1055/s-2003-41182 12905085

[B55] Dupuy O. Douzi W. Theurot D. Bosquet L. Dugué B. (2018). An evidence-based approach for choosing post-exercise recovery techniques to reduce markers of muscle damage, soreness, fatigue, and inflammation: a systematic review with meta-analysis. *Front. Physiol.* 9:403. 10.3389/fphys.2018.00403 29755363 PMC5932411

[B56] Edge J. Mündel T. Weir K. Cochrane D. J. (2009). The effects of acute whole body vibration as a recovery modality following high-intensity interval training in well-trained, middle-aged runners. *Eur. J. Appl. Physiol* 105 421-428. 10.1007/s00421-008-0919-z 19011891

[B57] Eriksson Crommert M. Lacourpaille L. Heales L. J. Tucker K. Hug F. (2015). Massage induces an immediate, albeit short-term, reduction in muscle stiffness. *Scand. J. Med. Sci. Sports* 25 e490-e496. 10.1111/sms.12341 25487283

[B58] Espí-López G. V. Serra-Añó P. Cuenca-Martínez F. Suso-Martí L. Inglés M. (2020). Comparison between classic and light touch massage on psychological and physical functional variables in athletes: a randomized pilot trial. *Int. J. Ther. Massage Bodywork* 13 30-37. 10.3822/ijtmb.v13i3.551 32922579 PMC7454233

[B59] Eyssautier F. Gibert G. (2018). *Device for Managing the Movements of a Robot, and Associated Treatment Robot.* U.S. Patent No Patent No FR3067957. Washington, DC: U.S. Patent and Trademark Office.

[B60] Fairall R. R. Cabell L. Boergers R. J. Battaglia F. (2017). Acute effects of self-myofascial release and stretching in overhead athletes with GIRD. *J. Bodywork Mov Ther.* 21 648-652. 10.1016/j.jbmt.2017.04.001 28750979

[B61] Fama B. J. Bueti D. R. (2011). *The Acute Effect of Self-Myofascial Release on Lower Extremity Plyometric Performance.* Limoges: IRFSS.

[B62] Farr T. Nottle C. Nosaka K. Sacco P. (2002). The effects of therapeutic massage on delayed onset muscle soreness and muscle function following downhill walking. *J. Sci. Med. Sport* 5 297-306. 10.1016/S1440-2440(02)80018-4 12585613

[B63] Field T. Grizzle N. Scafidi F. Abrams S. Richardson S. Kuhn C. (1996). Massage theraphy for infants of depressed mothers. *Infant Behav. Dev.* 19 107-112. 10.1016/S0163-6383(96)90048-X

[B64] Field T. Hernande-Reif M. Diego M. Schanberg S. Kuhn C. (2005). Cortisol decreases and serotonin and dopamine increase following massage therapy. *Int. J. Neurosci.* 115 1397-1413. 10.1080/00207450590956459 16162447

[B65] Fleckenstein J. Wilke J. Vogt L. Banzer W. (2017). Preventive and regenerative foam rolling are equally effective in reducing fatigue-related impairments of muscle function following exercise. *J. Sports Sci. Med.* 16 474-479. 29238246 PMC5721176

[B66] Garcia-Gutiérrez M. Guillén-Rogel P. Cochrane D. Marin P. (2018). Cross transfer acute effects of foam rolling with vibration on ankle dorsiflexion range of motion. *J. Musculoskeletal Neuronal Interact.* 18 262–267. PMC6016502 29855449

[B67] Gaullier J. (2015). *Effets du massage sportif sur la performance et la récupération entre croyances et preuves scientifiques.* Limeges: Institut Régional de Formation Sanitaire et Sociale du Limousin.

[B68] Ginszt M. Gawda P. Smołka J. Skublewska-Paszkowska M. Łukasik E. Paæko M. (2017). The immediat effect of self-myofascial release using a foam roller on electromyographic muscle activity. *Pol. J. Sports Med.* 33 209–213.

[B69] Golovin V. Samorukov A. Arkhipov M. Kocherevskaya L. (2018). Robotic restorative massage to increase working capacity. *Altern. Integr. Med.* 7:2 10.4172/2327-5162.1000261

[B70] Grabow L. Young J. D. Byrne J. M. Granacher U. Behm D. G. (2017). Unilateral rolling of the foot did not affect non-local range of motion or balance. *J. Sports Sci. Med.* 16 209-218. 28630574 PMC5465983

[B71] Grieve R. Barnett S. Coghill N. Cramp F. (2013). Myofascial trigger point therapy for triceps surae dysfunction: a case series. *Man. Ther.* 18 519-525. 10.1016/j.math.2013.04.004 23756031

[B72] Grieve R. Goodwin F. Alfaki M. Bourton A.-J. Jeffries C. Scott H. (2015). The immediate effect of bilateral self myofascial release on the plantar surface of the feet on hamstring and lumbar spine flexibility: a pilot randomised controlled trial. *J. Bodywork Mov. Ther.* 19 544-552. 10.1016/j.jbmt.2014.12.004 26118527

[B73] Guillot A. Kerautret Y. Queyrel F. Schobb W. Di Rienzo F. (2019). Foam rolling and joint distraction with elastic band training performed for 5-7 weeks respectively improve lower limb flexibility. *J. Sports Sci. Med.* 18 160–171. 30787664 PMC6370967

[B74] Guimberteau J. C. (2004). *Promenades sous la peau.* Amsterdam: Elsevier Masson.

[B75] Guimberteau J. C. Findley T. W. Kapandji A. I. Armstrong C. (2016). *L’architecture du corps humain vivant: Le monde extracellulaire, les cellules et le fascia révélés par l’endoscopie intratissulaire.* Available online at: https://books.google.fr/books?id=l9n4DAEACAAJ

[B76] Halperin I. Aboodarda S. J. Button D. C. Andersen L. L. Behm D. G. (2014). Roller massager improves range of motion of plantar flexor muscles without subsquent decreases in force parameters. *Int. J. Sports Phys. Ther.* 9 92-102. 24567860 PMC3924613

[B77] Han S. Lee Y. Lee D. (2017). The influence of the vibration form roller exercise on the pains in the muscles around the hip joint and the joint performance. *J. Phys. Ther. Sci.* 29 1844–1847. 10.1589/jpts.29.1844 29184303 PMC5684024

[B78] Hasegawa Y. Ootsuka T. Fukuda T. Arai F. Kawaguchi M. (2001). “A relaxation system adapting to user’s condition-identification of relationship between massage intensity and heart rate variability,” in *Proceedings of the IEEE International Conference on Robotics and Automation*, Brisbane, 3195-3200. 10.1109/ROBOT.2001.933110

[B79] Healey K. C. Hatfield D. L. Blanpied P. Dorfman L. R. Riebe D. (2014). The effects of myofascial release with foam rolling on performance. *J. Strength Condition. Res.* 28 61–68. 10.1519/jsc.0b013e3182956569 23588488

[B80] Hemmings B. Smith M. Graydon J. Dyson R. (2000). Effects of massage on physiological restoration, perceived recovery, and repeated sports performance. *Br. J. Sports Med.* 34:109. 10.1136/bjsm.34.2.109 10786866 PMC1724183

[B81] Hendricks S. Hill H. Hollander S. D. Lombard W. Parker R. (2019). Effects of foam rolling on performance and recovery: a systematic review of the literature to guide practitioners on the use of foam rolling. *J. Bodywork Mov. Ther.* 24 151-174. 10.1016/j.jbmt.2019.10.019 32507141

[B82] Hilbert J. E. Sforzo G. A. Swensen T. (2003). The effects of massage on delayed onset muscle soreness. *Br. J. Sports Med.* 37:72. 10.1136/bjsm.37.1.72 12547748 PMC1724592

[B83] Hinds T. McEwan I. Perkes J. Dawson E. Ball D. George K. (2004). Effects of massage on limb and skin blood flow after quadriceps exercise. *Med. Sci. Sports Exerc.* 36 1308–1313. 10.1249/01.mss.0000135789.47716.db 15292737

[B84] Hiraiwa Y. Ariji Y. Kise Y. Sakuma S. Kurita K. Ariji E. (2013). Efficacy of massage treatment technique in masseter muscle hardness: robotic experimental approach. *CRANIO^®^* 31 291-299. 10.1179/crn.2013.31.4.007 24308103

[B85] Hodgson D. D. Lima C. D. Low J. L. Behm D. G. (2018). Four weeks of roller massage training did not impact range of motion, pain pressure threshold, voluntary contractile properties or jump performance. *Int. J. Sports Phys. Ther.* 13 835-845. 10.26603/ijspt20180835 30276016 PMC6159503

[B86] Hodgson D. D. Quigley P. J. Whitten J. H. D. Reid J. C. Behm D. G. (2019). Impact of 10-minute interval roller massage on performance and active range of motion. *J. Strength Condition. Res.* 33 1512-1523. 10.1519/JSC.0000000000002271 29189581

[B87] Hotfiel T. Swoboda B. Krinner S. Grim C. Engelhardt M. Uder M. (2017). Acute effects of lateral thigh foam rolling on arterial tissue perfusion determined by spectral doppler and power doppler ultrasound. *J. Strength Cond. Res.* 31 893–900. 10.1519/jsc.0000000000001641 27749733

[B88] Hu L. Wang Y. Zhang J. Zhang J. Cui Y. Ma L. (2013). A massage robot based on chinese massage therapy. *Industr. Robot Int. J.* 40 158-172. 10.1108/01439911311297775

[B89] Hughes G. A. Ramer L. M. (2019). Duration of myofascial rolling for optimal recovery, range of motion, and performance: a systematic review of the litterature. *Int. J. Sports Phys. Ther.* 14 845-859. 10.26603/ijspt20190845 31803517 PMC6878859

[B90] Ishii H. Koga H. Obokawa Y. Solis J. Takanishi A. Katsumata A. (2009). Development and experimental evaluation of oral rehabilitation robot that provides maxillofacial massage to patients with oral disorders. *Int. J. Robot. Res.* 28 1228-1239. 10.1177/0278364909104295

[B91] Iwamoto K. Mizukami M. Asakawa Y. Yoshio M. Ogaki R. Takemura M. (2016). Effects of friction massage of the popliteal fossa on dynamic changes in muscle oxygenation and ankle flexibility. *J. Phys. Ther. Sci.* 28 2713–2716. 10.1589/jpts.28.2713 27821920 PMC5088111

[B92] Jafarnezhadgero A. A. Majlesi M. Etemadi H. Robertson D. G. E. (2018). Rehabilitation improves walking kinematics in children with a knee varus: randomized controlled trial. *Ann. Phys. Rehabil. Med.* 61 125-134. 10.1016/j.rehab.2018.01.007 29476933

[B93] Jakeman J. R. Byrne C. Eston R. G. (2010). Efficacy of lower limb compression and combined treatment of manual massage and lower limb compression on symptoms of exercise-induced muscle damage in women. *J. Strength Condition. Res.* 24 3157–3165. 10.1519/jsc.0b013e3181e4f80c 20940646

[B94] Jay K. Sundstrup E. Søndergaard S. D. Behm D. Brandt M. Særvoll C. A. (2014). Specific and cross over effects of massage for muscle soreness: Randomized controlled trial. *Int. J. Sports Phys. Ther.* 9 82-91. 24567859 PMC3924612

[B95] Jeong Y. Park J. Yu J. Lee S. Ha J. Choo Y. (2019). Immediate effects of release ball massage and self-stretching exercise on hamstring’s temperature, range of motion and strength in 20’s women. *J. Int. Acad. Phys. Ther. Res.* 10 1739-1745. 10.20540/JIAPTR.2019.10.1.1739

[B96] Jimenez G. F. C. (2019). *Sistema robótico para tratamientos fisioterapéuticos, mediante robot manipulador colaborativo y aire comprimido.* U.S. Patent No ES1222864Y. Washington, DC: U.S. Patent and Trademark Office.

[B97] Joshi D. G. Balthillaya G. Prabhu A. (2018). Effect of remote myofascial release on hamstring flexibility in asymptomatic individuals – a randomized clinical trial. *J. Bodywork Mov. Ther.* 22 832-837. 10.1016/j.jbmt.2018.01.008 30100320

[B98] Jourdain C. (2015). “Étude des effets d’une séance de massage hebdomadaire sur la perception des douleurs musculaires et des perceptions de la récupération globale chez des jeunes athlètes de haut niveau,” in *Proceedings of the Direction Régionale de la Jeuensse, des Sports et de la Cohésion sociale*, Paris.

[B99] Jung J. Choi W. Lee Y. Kim J. Kim H. Lee K. (2017). Immediate effect of self-myofascial release on hamstring flexibility. *Phys. Ther. Rehabil. Sci.* 6 45-51. 10.14474/ptrs.2017.6.1.45

[B100] Kaada B. Torsteinb O. (1989). Increase of plasma β-endorphins in connective tissue massage. *Gen. Pharmacol.Vascu. Syst.* 20 487-489. 10.1016/0306-3623(89)90200-0 2526775

[B101] Kalén A. Pérez-Ferreirós A. Barcala-Furelos R. Fernández-Méndez M. Padrón-Cabo A. Prieto J. A. (2017). How can lifeguards recover better? A cross-over study comparing resting, running, and foam rolling. *Am. J. Emerg. Med.* 35:5. 10.1016/j.ajem.2017.06.028 28651888

[B102] Kalichman L. Ben David C. (2017). Effect of self-myofascial release on myofascial pain, muscle flexibility, and strength: a narrative review. *J. Bodywork Mov. Ther.* 21 446-451. 10.1016/j.jbmt.2016.11.006 28532889

[B103] Kargarfard M. Lam E. T. C. Shariat A. Shaw I. Shaw B. S. Tamrin S. B. M. (2016). Efficacy of massage on muscle soreness, perceived recovery, physiological restoration and physical performance in male bodybuilders. *J. Sports Sci.* 34 959-965. 10.1080/02640414.2015.1081264 26334128

[B104] Kelly S. Beardsley C. (2016). Specific and cross-over effects of foam rolling on ankle dorsiflexion range of motion. *Int. J. Sports Phys. Ther.* 11 544-551. 27525179 PMC4970845

[B105] Kett A. R. Sichting F. (2020). Sedentary behaviour at work increases muscle stiffness of the back: why roller massage has potential as an active break intervention. *Appl. Ergon.* 82:102947. 10.1016/j.apergo.2019.102947 31514046

[B106] Killen B. S. Zelizney K. L. Ye X. (2018). Crossover effects of unilateral static stretching and foam rolling on contralateral hamstring flexibility and strength. *J. Sport Rehabil.* 28 1-27. 10.1123/jsr.2017-0356 29543123

[B107] Kim K. Park S. Goo B.-O. Choi S.-C. (2014). Effect of self-myofascial release on reduction of physical stress: a pilot study. *J. Phys. Ther. Sci.* 26 1779-1781. 10.1589/jpts.26.1779 25435699 PMC4242954

[B108] Kim Y. Hong Y. Park H.-S. (2019). A soft massage tool is advantageous for compressing deep soft tissue with low muscle tension: therapeutic evidence for self-myofascial release. *Complement. Ther. Med* 43 312-318. 10.1016/j.ctim.2019.01.001 30935551

[B109] Koga H. Usuda Y. Matsuno M. Ogura Y. Ishii H. Solis J. (2008). “Development of the oral rehabilitation robot WAO-1,” in *Proceedings of the 2008 2nd IEEE RAS & EMBS International Conference on Biomedical Robotics and Biomechatronics*, Scottsdale, AZ, 556–561.

[B110] Kong P. W. Chua Y. H. Kawabata M. Burns S. F. Cai C. (2018). Effect of post-exercise massage on passive muscle stiffness measured using myotonometry—A double-blind study. *J. Sports Sci. Med.* 17 599-606. 30479528 PMC6243630

[B111] Kyranoudis A. Arsenis S. Ispyrlidis I. Chatzinikolaou A. Gourgoulis V. Kyranoudis E. (2019). The acute effects of combined foam rolling and static stretching program on hip flexion and jumping ability in soccer players. *J. Phys. Educ. Sport* 19 1164-1172. 10.7752/jpes.2019.02169

[B112] Lastova K. Nordvall M. Walters-Edwards M. Allnutt A. Wong A. (2018). Cardiac autonomic and blood pressure responses to an acute foam rolling session. *J. Strength Condition. Res.* 32 2825–2830. 10.1519/jsc.0000000000002562 29570571

[B113] Lau W. Y. Nosaka K. (2011). Effect of vibration treatment on symptoms associated with eccentric exercise-induced muscle damage. *Am. J. Phys. Med. Rehabil.* 90 648–657. 10.1097/phm.0b013e3182063ac8 21273897

[B114] Le Gal J. Begon M. Gillet B. Rogowski I. (2018). Effects of self-myofascial release on shoulder function and perception in adolescent tennis players. *J. Sport Rehabil.* 27 1-6. 10.1123/jsr.2016-0240 28952852

[B115] Lee C.-H. Lai C.-L. Sung Y.-H. Lai M. Y. Lin C.-Y. Lin L.-Y. (2017). Comparing effects between music intervention and aromatherapy on anxiety of patients undergoing mechanical ventilation in the intensive care unit: a randomized controlled trial. *Qual. Life Res.* 26 1819-1829. 10.1007/s11136-017-1525-5 28236262

[B116] Lee H.-I. Lim B.-O. (2018). Effects of self myofascial release, elastic band, and stretching exercises on lower extremity alignment and gait in female genu varum. *Korean J. Sport Biomech.* 28 207-211. 10.5103/KJSB.2018.28.4.207

[B117] Lefranc M. Peltier J. (2016). Evaluation of the ROSATM Spine robot for minimally invasive surgical procedures. *Exp. Rev. Med. Dev.* 13 899-906. 10.1080/17434440.2016.1236680 27649314

[B118] Leivadi S. Hernandez-Reif M. Field T. O’Rourke M. D’Arienzo S. Lewis D. (1999). Massage therapy and relaxation effects on university dance students. *J. Dance Med. Sci.* 3, 108–112.

[B119] Lim J.-H. Park C.-B. (2019). The immediate effects of foam roller with vibration on hamstring flexibility and jump performance in healthy adults. *J. Exerc. Rehabil.* 15 50-54. 10.12965/jer.1836560.280 30899736 PMC6416504

[B120] Lima C. R. Martins D. F. Reed W. R. (2020). Physiological responses induced by manual therapy in animal models: a scoping review. *Front. Neurosci.* 14:430. 10.3389/fnins.2020.00430 32457570 PMC7227122

[B121] Lopez E. Kwok K. Payne C. J. Giataganas P. Yang G. (2013). “Implicit Active Constraints for robot-assisted arthroscopy,” in *Proceedings of the 2013 IEEE International Conference on Robotics and Automation*, Karlsruhe, 5390-5395. 10.1109/ICRA.2013.6631350 PMC3988876 24748994

[B122] Luo R. C. Chang C. C. (2011). “Electromyographic evaluation of therapeutic massage effect using multi-finger robot hand,” in *Proceedings of the 2011 IEEE International Conference on Robotics and Automation*, Shanghai, 2431-2436. 10.1109/ICRA.2011.5980147

[B123] Luo R. C. Hsu C.-W. Chen S.-Y. (2016). “Electroencephalogram signal analysis as basis for effective evaluation of robotic therapeutic massage,” in *Proceedings of the 2016 IEEE/RSJ International Conference on Intelligent Robots and Systems (IROS)*, Daejeon, 2940-2945. 10.1109/IROS.2016.7759455

[B124] MacDonald G. Z. Button D. C. Drinkwater E. J. Behm D. G. (2014). Foam rolling as a recovery tool after an intense bout of physical activity. *Med. Sci. Sports Exerc.* 46 131–142. 10.1249/mss.0b013e3182a123db 24343353

[B125] MacDonald G. Z. Penney M. D. H. Mullaley M. E. Cuconato A. L. Drake C. D. J. Behm D. G. (2013). An acute bout of self-myofascial release increases range of motion without a subsequent decrease in muscle activation or force. *J. Strength Condition. Res.* 27 812–821. 10.1519/jsc.0b013e31825c2bc1 22580977

[B126] Macgregor L. J. Fairweather M. M. Bennett R. M. Hunter A. M. (2018). The effect of foam rolling for three consecutive days on muscular efficiency and range of motion. *Sports Med. Open* 4:26. 10.1186/s40798-018-0141-4 29884972 PMC5993692

[B127] Mackin C. (2017). *Robotic Massage Machine and Method of Use.* U.S. Patent No US2017266077. Washington, DC: U.S. Patent and Trademark Office.

[B128] Madoni S. N. Costa P. B. Coburn J. W. Galpin A. J. (2018). Effects of foam rolling on range of motion, peak torque, muscle activation, and the hamstrings-to-quadriceps strength ratios. *J. Strength Condition. Res.* 32 1821–1830. 10.1519/jsc.0000000000002468 29401195

[B129] Mancinelli C. A. Davis D. S. Aboulhosn L. Brady M. Eisenhofer J. Foutty S. (2006). The effects of massage on delayed onset muscle soreness and physical performance in female collegiate athletes. *Phys. Ther. Sport* 7 5-13. 10.1016/j.ptsp.2005.10.004

[B130] Martínez-Cabrera F. I. Núñez-Sánchez F. J. (2016). Acute effect of a foam roller on the mechanical properties of the rectus femoris based on tensiomyography in soccer players. *Int. J. Hum. Mov. Sports Sci.* 4 26–32. 10.13189/saj.2016.040203

[B131] Mazzei B. G. (2019). *Different effects of static and vibrating foam rollers on ankle plantar flexion flexibility and neuromuscular activation.* Thesis in Exercise Science. Statesboro: Georgia Southern University.

[B132] McKechnie G. J. Young W. B. Behm D. G. (2007). Acute effects of two massage techniques on ankle Joint flexibility and power of the plantar flexors. *J. Sports Sci. Med.* 6 498-504. 24149484 PMC3794491

[B133] Mezencevová V. Torok J. Czánová T. Zajac J. (2017). Endermologie new aproach in the medicine treatment. *Technol. Eng.* 14 10.1515/teen-2017-0008

[B134] Mikesky A. E. Bahamonde R. E. Stanton K. Alvey T. Fitton T. (2002). Acute effects of the stick on strength, power, and flexibility. *J. Strength Condition. Res.* 16 446–450. 10.1519/00124278-200208000-00017 12173961

[B135] Miller J. K. Rockey A. M. (2006). Foam rollers show no increase in the flexibility of the hamstring muscle group. *J. Undergraduate Res.* 9 9–14.

[B136] Mohr A. R. Long B. C. Goad C. L. (2014). Effect of foam rolling and static stretching on passive hip-flexion range of motion. *J. Sport Rehabil.* 23 296-299. 10.1123/JSR.2013-0025 24458506

[B137] Monteiro E. R. Cavanaugh M. T. Frost D. M. Novaes J. D. S. (2017). Is self-massage an effective joint range-of-motion strategy? A pilot study. *J. Bodywork Mov. Ther.* 21 223-226. 10.1016/j.jbmt.2016.10.003 28167184

[B138] Monteiro E. R. da Silva Novaes J. Cavanaugh M. T. Hoogenboom B. J. Steele J. Vingren J. L. (2019). Quadriceps foam rolling and rolling massage increases hip flexion and extension passive range-of-motion. *J. Bodywork Mov. Ther.* 23 575–580. 10.1016/j.jbmt.2019.01.008 31563372

[B139] Monteiro E. R. Škarabot J. Vigotsky A. D. Brown A. F. Gomes T. M. Novaes J. D. S. (2017a). Acute effect of different self-massage volumes on the FMSTM overhead deep squat performance. *Int. J. Sports Phys. Ther.* 12 94-104. 28217420 PMC5294950

[B140] Monteiro E. R. Škarabot J. Vigotsky A. D. Brown A. F. Gomes T. M. Novaes J. D. S. (2017b). Maximum repetition performance after different antagonist foam rolling volumes in the inter-set rest period. *Int. J. Sports Phys. Ther.* 12 76-84. PMC5294949 28217418

[B141] Moraska A. (2007). Therapist education impacts the massage effect on postrace muscle recovery. *Med. Sci. Sports Exerc.* 39 34–37. 10.1249/01.mss.0000240320.16029.d2 17218881

[B142] Mori H. Ohsawa H. Tanaka T. H. Taniwaki E. Leisman G. Nishijo K. (2004). Effect of massage on blood flow and muscle fatigue following isometric lumbar exercise. *Med. Sci. Monit.* 10 173–178. 15114265

[B143] Murali S. Shanmugam S. V. Prasaad G. A. Kumar M. S. Manoharan C. Devadasan S. R. (2014). Fatigue mitigation through the optimization of ergonomic positional parameters in massage therapy using virtual instrumentation. *Int. J. Adv. Manufact. Technol.* 70 173-184. 10.1007/s00170-013-5259-4

[B144] Murray A. M. Jones T. W. Horobeanu C. Turner A. P. Sproule J. (2016). Sixty seconds of foam rolling does not affect functional flexibility or change muscle temperature in adolescent athletes. *Int. J. Sports Phys. Ther.* 11 765-776. 27757289 PMC5046970

[B145] Myers T. W. (2013). *Anatomy Trains E-Book: Myofascial Meridians for Manual and Movement Therapists.* Amsterdam: Elsevier Health Sciences.

[B146] Naderi A. Rezvani M. H. Degens H. (2019). Foam rolling and muscle and joint proprioception after exercise-induced muscle damage. *J. Athletic Train.* 55 58-64. 10.4085/1062-6050-459-18 31855077 PMC6961644

[B147] Nakano H. Kodama T. Ueda T. Mori I. Tani T. Murata S. (2019). Effect of hand and foot massage therapy on psychological factors and EEG activity in elderly people requiring long-term care: a randomized cross-over study. *Brain Sci.* 9:54. 10.3390/brainsci9030054 30836612 PMC6468439

[B148] Nissim E. (2001). *Human Touch Massager.* U.S. Patent No US2001014781. Washington, DC: U.S. Patent and Trademark Office.

[B149] Obokawa Y. Solis J. Ishii H. Koga H. Takanishi A. Katsumata A. (2009). “Clinical massage therapy with the oral-rehabilitation robot in patients with temporomandibular joint disorders,” in *Proceedings of the 2009 9th International Conference on Information Technology and Applications in Biomedicine*, Larcana, 1–4.

[B150] Ogai R. Yamane M. Matsumoto T. Kosaka M. (2008). Effects of petrissage massage on fatigue and exercise performance following intensive cycle pedaling. *Br. J. Sports Med* 42 834-838. 10.1136/bjsm.2007.044396 18385196

[B151] Okamoto T. Masuhara M. Ikuta K. (2014). Acute effects of self-myofascial release using a foam roller on arterial function. *J. Strength Condition. Res.* 28 69–73. 10.1519/jsc.0b013e31829480f5 23575360

[B152] Oranchuk D. J. Flattery M. R. Robinson T. L. (2019). Superficial heat administration and foam rolling increase hamstring flexibility acutely; with amplifying effects. *Phys. Ther. Sport* 40 213-217. 10.1016/j.ptsp.2019.10.004 31605900

[B153] Patel D. G. Vyas N. J. Sheth M. S. (2016). Immediate effect of application of bilateral self myo-fascial release on the plantar surface of the foot on hamstring and lumbar spine flexibility: a quasi experimental study. *Int. J. Ther. Appl.* 32 94-99. 10.20530/IJTA_32_94-99

[B154] Pathania T. Muragod A. R. (2019). Comparative effect of foam roller and M2T blade on hamstring flexibility in elderly population: a randomized control trial. *Indian J. Health Sci. Biomed. Res.* 12:160 10.4103/kleuhsj.kleuhsj_118_18

[B155] Patole S. Sayyad S. Palekar T. J. (2019). To compare the effect of myofascial release technique versus foam rolling on hamstring spasticity in spastic Diplegia: pilot study. *Int. J. Yoga Physiother. Phys. Educ.* 4 65–70.

[B156] Paz G. Maia M. Santana H. Silva J. Lima V. (2017). Electromyographic analysis of muscles activation during sit-and-reach test adopting self-myofascial release with foam rolling versus traditional warm. *J. Athlet. Enhance.* 4 26-28. 10.4172/2324-9080.1000248

[B157] Peacock C. A. Krein D. D. Antonio J. Sanders G. J. Silver T. A. Colas M. (2015). Comparing acute bouts of sagittal plane progression foam rolling vs. Frontal plane progression foam rolling. *J. Strength Condition. Res.* 29:2310–2315. 10.1519/jsc.0000000000000867 25647651

[B158] Peacock C. A. Krein D. D. Silver T. A. Sanders G. J. Von Carlowitz K.-P. A. (2014). An acute bout of self-myofascial release in the form of foam rolling improves performance testing. *Int. J. Exerc. Sci.* 7:202. 10.70252/DTPM9041 PMC4831860 27182404

[B159] Pearcey G. E. P. Bradbury-Squires D. J. Kawamoto J.-E. Drinkwater E. J. Behm D. G. Button D. C. (2015). Foam rolling for delayed-onset muscle soreness and recovery of dynamic performance measures. *J. Athletic Train.* 50 5-13. 10.4085/1062-6050-50.1.01 25415413 PMC4299735

[B160] Peng C.-C. Hwang T.-S. Lin C.-J. Wu Y.-T. Chang C.-Y. Huang J.-B. (2010). “Development of intelligent massage manipulator and reconstruction of massage process path using image processing technique,” in *Proceedings of the 2010 IEEE Conference on Robotics, Automation and Mechatronics*, Singapore, 551-556. 10.1109/RAMECH.2010.5513135

[B161] Petrescu R. V. Aversa R. Apicella A. Petrescu F. I. (2016). Future medicine services robotics. *Am. J. Eng. Appl. Sci.* 9 1062-1087. 10.3844/ajeassp.2016.1062.1087

[B162] Phillips J. Diggin D. King D. L. Sforzo G. A. (2018). Effect of varying self-myofascial eelease duration on subsequent athletic performance. *J. Strength Condition. Res.* [Epub ahead of print], 10.1519/JSC.0000000000002751 30024480

[B163] Pinar S. Kaya F. Bicer B. Erzeybek M. Cotuk H. (2012). Different recovery methods and muscle performance after exhausting exercise: comparison of the effects of electrical muscle stimulation and massage. *Biol. Sport* 29 269-275. 10.5604/20831862.1019664 24868117 PMC4033060

[B164] Poppendieck W. Wegmann M. Ferrauti A. Kellmann M. Pfeiffer M. Meyer T. (2016). Massage and performance recovery: a meta-analytical review. *Sports Med.* 46 183-204. 10.1007/s40279-015-0420-x 26744335

[B165] Qiu C. Zhang Y. Kai L. (2019). *Massage apparatus.* *https://worldwide.espacenet.com/publicationDetails/biblio?FT=Danddate=20190411andDB=andlocale=fr_EPandCC=WOandNR=2019070198A1andKC=A1andND=1*.

[B166] Rapaport M. H. Schettler P. Bresee C. (2010). A preliminary study of the effects of a single session of swedish massage on hypothalamic–pituitary–adrenal and immune function in normal individuals. *J. Alternat. Compl. Med* 16 1079-1088. 10.1089/acm.2009.0634 20809811 PMC3107905

[B167] Rey E. Padrón-Cabo A. Costa P. B. Barcala-Furelos R. (2017). The effects of foam rolling as a recovery tool in professional soccer players. *J. Strength Condition. Res.* 33 2194-2201. 10.1519/JSC.0000000000002277 29016479

[B168] Richards K. (1998). Effect of a back massage and relaxation intervention on sleep in critically ill patients. *Am. J. Crit. Care* 7 288. 10.4037/ajcc1998.7.4.288 9656043

[B169] Rinder A. N. Sutherland C. J. (1995). An investigation of the effects of massage on quadriceps performance after exercise fatigue. *Compl. Ther. Nurs. Midwif.* 1 99-102. 10.1016/S1353-6117(05)80048-4 9456718

[B170] Rivera M. Eberman L. Games K. Powden C. J. (2019). Comparison of myofascial release techniques on pectoralis minor length, glenohumeral total arc of motion, and skin temperature: a pilot study. *J. Sport Rehabil.* 24 1-5. 10.1123/jsr.2018-0130 30526261

[B171] Robertson A. Watt J. M. Galloway S. D. R. (2004). Effects of leg massage on recovery from high intensity cycling exercise. *Br. J. Sports Med.* 38:173. 10.1136/bjsm.2002.003186 15039254 PMC1724761

[B172] Romero-Moraleda B. González-García J. Cuéllar-Rayo Á Balsalobre-Fernández C. Muñoz-García D. Morencos E. (2019). Effects of vibration and non-vibration foam rolling on recovery after exercise with induced muscle damage. *J. Sports Sci. Med.* 18 172-180. 30787665 PMC6370959

[B173] Romero-Moraleda B. La Touche R. Lerma-Lara S. Ferrer-Peña R. Paredes V. Peinado A. B. (2017). Neurodynamic mobilization and foam rolling improved delayed-onset muscle soreness in a healthy adult population: a randomized controlled clinical trial. *PeerJ* 5:18. 10.7717/peerj.3908 29043110 PMC5642244

[B174] Rossettini G. Rondoni A. Schiavetti I. Tezza S. Testa M. (2016). Prevalence and risk factors of thumb pain in Italian manual therapists: an observational cross-sectional study. *Work* 54 159-169. 27061697 10.3233/WOR-162289

[B175] Sağiroğlu Ý (2017). Acute effect of applied local vibration during foam roller exercices on lower extremety explosive strength and flexibility performance. *Eur. J. Phys. Educ. Sport Sci.* 3 20-30. 10.5281/zenodo.89696

[B176] Saitou K. Tokunaga M. Yoshino D. Sakitani N. Maekawa T. Ryu Y. (2018). Local cyclical compression modulates macrophage function in situ and alleviates immobilization-induced muscle atrophy. *Clin. Sci.* 132 2147-2161. 10.1042/CS20180432 30209036

[B177] Sakitani N. Maekawa T. Saitou K. Suzuki K. Murase S. Tokunaga M. (2019). Application of consistent massage-like perturbations on mouse calves and monitoring the resulting intramuscular pressure changes. *JoVE* 151:e59475. 10.3791/59475 31589203

[B178] Schleip R. (2003b). Fascial plasticity – a new neurobiological explanation Part 2. *J. Bodywork Mov. Ther.* 7 104-116. 10.1016/S1360-8592(02)00076-1

[B179] Schleip R. (2003a). Fascial plasticity – a new neurobiological explanation: Part 1. *J. Bodywork Mov. Ther.* 7 11-19. 10.1016/S1360-8592(02)00067-0

[B180] Schroeder A. N. Best T. M. (2015). Is self myofascial release an effective preexercise and recovery strategy? A literature review. *Curr. Sports Med. Rep.* 14 200–208. 10.1249/jsr.0000000000000148 25968853

[B181] Schroeder J. Renk V. Braumann K.-M. Hollander K. (2017). Acute foam rolling effects on contractile properties of the m. Biceps femoris. *German J. Exerc. Sport Res.* 47 294-300. 10.1007/s12662-017-0467-y

[B182] Sharp V. (2012). *A Comparative Study Between Self Myofascial Release and Emmett Technique Effectiveness in the Management of fascial (iliotibial band) Tightness. Queen University Dissertation.* Belfast: Stranmillis University College.

[B183] Sharpe P. A. Williams H. G. Granner M. L. Hussey J. R. (2007). A randomised study of the effects of massage therapy compared to guided relaxation on well-being and stress perception among older adults. *Compl. Ther. Med.* 15 157-163. 10.1016/j.ctim.2007.01.004 17709060

[B184] Škarabot J. Beardsley C. Štirn I. (2015). Comparing the effect of self-myofascial release with static stretching on ankle range-of-motion in adolescent athletes. *Int. J. Sports Phys. Ther.* 10 203-212. 25883869 PMC4387728

[B185] Skinner B. Moss R. Hammond L. (2020). A systematic review and meta-analysis of the effects of foam rolling on range of motion, recovery and markers of athletic performance. *J. Bodywork Mov. Ther.* 24 105–122. 10.1016/j.jbmt.2020.01.007 32825976

[B186] Smith J. C. Washell B. R. Aini M. F. Brown S. Hall M. C. (2019). Effects of static stretching and foam rolling on ankle dorsiflexion range of motion. *Med. Sci. Sports Exerc.* 51 1752-1758. 10.1249/mss.0000000000001964 30817716

[B187] Smith L. Keating M. N. Holbert D. Spratt D. J. McCammon M. R. Smith S. S. (1994). The effects of athletic massage on delayed onset muscle soreness, creatine kinase, and neutrophil count: a preliminary report. *J. Orthop. Sports Phys. Ther.* 19 93-99. 10.2519/jospt.1994.19.2.93 8148868

[B188] Solis J. Obokawa Y. Ishii H. Koga H. Takanishi A. Katsumata A. (2009). “Development of oral rehabilitation robot WAO-1R designed to provide various massage techniques,” in *Proceedings of the IEEE International Conference on Rehabilitation Robotics*, (Kyoto: IEEE), 457-462.

[B189] Somers K. Aune D. Horten A. Kim J. Rogers J. (2019). Acute effects of gastrocnemius/soleus self-myofascial release versus dynamic stretching on closed-chain dorsiflexion. *J. Sport Rehabil.* 29 1-28. 10.1123/jsr.2018-0199 30747565

[B190] Standley R. A. Miller M. G. Binkley H. (2010). Massage’s effect on injury, recovery, and performance: a review of techniques and treatment parameters. *StrengthCondition. J.* 32 64–67. 10.1519/ssc.0b013e3181c33918

[B191] Stroiney D. A. Mokris R. L. Hanna G. R. Ranney J. D. (2020). Examination of self-myofascial release vs. Instrument-assisted soft-Ttssue mobilization techniques on vertical and horizontal power in recreational athletes. *J. Strength Condition. Res.* 34 79-88. 10.1519/JSC.0000000000002628 29742744

[B192] Su H. Chang N.-J. Wu W.-L. Guo L.-Y. Chu I.-H. (2017). Acute effects of foam rolling, static stretching, and dynamic stretching during warm-ups on muscular flexibility and strength in young adults. *J. Sport Rehabil.* 26 469-477. 27736289 10.1123/jsr.2016-0102

[B193] Sullivan K. M. Silvey D. B. Button D. C. Behm D. G. (2013). Roller-massager application to the hamstrings increases sit-and-reach range of motion within five to ten seconds without performance impairment. *Int. J. Sports Phys. Ther.* 8 228-236. 23772339 PMC3679629

[B194] Suzuki M. Tatsumi A. Otsuka T. Kikuchi K. Mizuta A. Makino K. (2010). Physical and psychological effects of 6-week tactile massage on elderly patients with severe dementia. *Am. J. Alzheimers Dis. Other Dement.* 25 680-686. 10.1177/1533317510386215 21131675 PMC10845700

[B195] Takanishi A. Katsumata A. Koga H. Ishii H. Solis J. Obokawa Y. (2009). *Massage Robot and Control Program Thereof.* U.S. Patent No WO2009118933. Washington, DC: U.S. Patent and Trademark Office.

[B196] Takanishi A. Katsumata A. Usuda Y. Koga H. Matsuno M. Ogura Y. (2008). *Massage Robot, Control Program Therefor, and Robot for Specifying Portion of Human Body.* U.S. Patent No WO2008041457. Washington, DC: U.S. Patent and Trademark Office.

[B197] Thistlethwaite J. Vonderhaar R. Hockenberry K. Rindler L. Cayot T. Nelson B. (2016). The effects of foam-rolling on femoral endothelial function. *Med. Sci. Sports Exerc.* 48:1070. 10.1249/01.mss.0000488219.26597.5b 30958151

[B198] Thomson D. Gupta A. Arundell J. Crosbie J. (2015). Deep soft-tissue massage applied to healthy calf muscle has no effect on passive mechanical properties: A randomized, single-blind, cross-over study. *BMC Sports Sci. Med. Rehabil.* 7:21. 10.1186/s13102-015-0015-8 26396740 PMC4578668

[B199] Tiidus P. Shoemaker J. (1995). Effleurage massage, muscle blood flow and long-term post-exercise strength recovery. *Int. J. Sports Med.* 16 478–483. 10.1055/s-2007-973041 8550258

[B200] Vigotsky A. D. Bruhns R. P. (2015). The role of descending modulation in manual therapy and its analgesic implications: a narrative review. *Pain Res. Treat.* 2015 1-11. 10.1155/2015/292805 26788367 PMC4695672

[B201] Vigotsky A. D. Lehman G. J. Contreras B. Beardsley C. Chung B. Feser E. H. (2015). Acute effects of anterior thigh foam rolling on hip angle, knee angle, and rectus femoris length in the modified Thomas test. *PeerJ* 3:e1281. 10.7717/peerj.1281 26421244 PMC4586805

[B202] Viitasalo J. T. Niemelä K. Kaappola R. Korjus T. Levola M. Mononen H. V. (1995). Warm underwater water-jet massage improves recovery from intense physical exercise. *Eur. J. Appl. Physiol. Occupat. Physiol.* 71 431-438. 10.1007/BF00635877 8565975

[B203] Visconti L. Capra G. Carta G. Forni C. Janin D. (2015). Effect of massage on DOMS in ultramarathon runners: a pilot study. *J. Bodywork Mov. Ther.* 19 458-463. 10.1016/j.jbmt.2014.11.008 26118518

[B204] Weerapong P. Hume P. A. Kolt G. S. (2005). The mechanisms of massage and effects on performance, muscle recovery and injury prevention. *Sports Med.* 35 235-256. 10.2165/00007256-200535030-00004 15730338

[B205] White G. E. West S. L. Caterini J. E. Battista A. P. D. Rhind S. G. Wells G. D. (2020). Massage therapy modulates inflammatory mediators following sprint exercise in healthy male athletes. *J. Funct. Morphol. Kinesiol.* 5:9 10.3390/jfmk5010009 PMC7739334 33467225

[B206] Wiewelhove T. Döweling A. Schneider C. Hottenrott L. Meyer T. Kellmann M. (2019). A meta-analysis of the effects of foam rolling on performance and recovery. *Front. Physiol.* 10:376. 10.3389/fphys.2019.00376 31024339 PMC6465761

[B207] Willems M. E. Hale T. Wilkinson C. S. (2009). Effect of manual massage on muscle-specific soreness and single leg jump performance after downhill treadmill walking. *Med. Sport.* 13 61–66. 10.2478/v10036-009-0011-8

[B208] Williams W. Selkow N. M. (2019). Self-myofascial release of the superficial back line improves sit-and-reach distance. *J. Sport Rehabil.* 1 1–5. 10.1123/jsr.2018-0306 30860410

[B209] Wiltshire E. V. Poitras V. Pak M. Hong T. Rayner J. Tschakovsky M. E. (2010). Massage impairs postexercise muscle blood flow and” lactic acide removal. *Med. Sci. Sports Exerc.* 42 1062–1071. 19997015 10.1249/MSS.0b013e3181c9214f

[B210] Xiaoqin Y. Yonggen X. (2010). “Design and simulation of Chinese massage robot based on parallel mechanism,” in *Proceedings of the 2010 International Conference on Mechanic Automation and Control Engineering*, 2512-2515. 10.1109/MACE.2010.5535326

[B211] Yang J. Chen S. Hsieh C.-L. Lin J. (2012). Effects and predictors of shoulder muscle massage for patients with posterior shoulder tightness. *BMC Musculoskelet. Disord.* 13:46. 10.1186/1471-2474-13-46 22449170 PMC3339516

[B212] Ye X. Killen B. S. Zelizney K. L. Miller W. M. Jeon S. (2019). Unilateral hamstring foam rolling does not impair strength but the rate of force development of the contralateral muscle. *PeerJ* 7:e7028. 10.7717/peerj.7028 31179197 PMC6545114

[B213] Zainuddin Z. Newton M. Sacco P. Nosaka K. (2005). Effects of massage on delayed-onset muscle soreness, swelling, and recovery of muscle function. *J. Athlet. Train.* 40 174-180. PMC1250256 16284637

[B214] Zelikovski A. Kaye C. L. Fink G. Spitzer S. A. Shapiro Y. (1993). The effects of the modified intermittent sequential pneumatic device (MISPD) on exercise performance following an exhaustive exercise bout. *Br. J. Sports Med.* 27:255. 10.1136/bjsm.27.4.255 8130964 PMC1332015

